# Polymers and light: a love–hate relationship

**DOI:** 10.1039/d5sc00997a

**Published:** 2025-03-17

**Authors:** M. A. Sachini N. Weerasinghe, Tochukwu Nwoko, Dominik Konkolewicz

**Affiliations:** a Department of Chemistry and Biochemistry, Miami University 651 E High St Oxford OH 45056 USA d.konkolewicz@miamioh.edu

## Abstract

The study of the interaction between polymers and light has significantly bloomed over the past few years in various fundamental research and applications. The relationship between polymers and light can be beneficial (we refer to this as “love”) or be destructive (we refer to this as “hate”). It is important to understand the nature of both these love and hate relationships between polymers and light to apply these concepts in various future systems, to surpass performance of existing materials, or to mitigate some problems associated with polymers. Therefore, this perspective highlights both the photophilic (*e.g.*, photopolymerization, rate modulation, temporal/spatial control, drug delivery, waste management, photo functionalization, and photo-enhanced depolymerization) and photophobic (*e.g.*, photodegradation, discoloration, optical density, and loss of functionality) nature of polymers.

## Abstract 2

The interaction of light with polymers can be positive, where light enhances polymerization or performance, but it can also be negative towards the targeted properties. Positive and negative interactions between polymers and light are highlighted.

## Graphical abstract

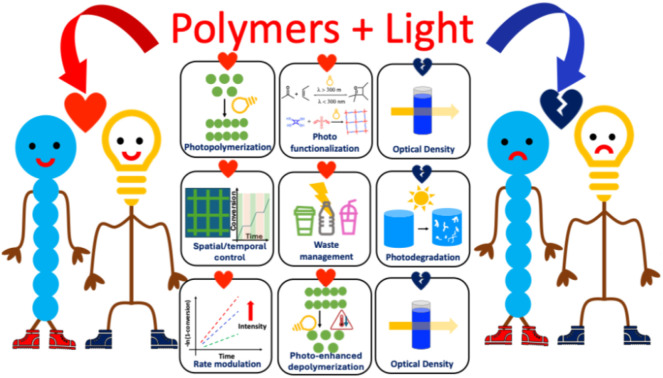

## Introduction

Polymers have been interacting with light for millions of years, both directly and indirectly.^1^ The energetic origin of all biopolymers including cellulose, starch, lignin, proteins, *etc.* is sunlight.^2^ Certain other biomacromolecules have unique interactions with light, such as the proteins clusters in photosynthesis and green fluorescent protein.^3^ However, light can also induce changes in materials and degradation of biopolymers, including aging of wood,^4^ UV-induced damage and pathogenesis in skin,^5^ and increase the risk of peptide degradation,^6^ among others. This interplay between light's ability to enhance biopolymers but also cause destruction is replicated in synthetic polymers.

**Fig. 1:**
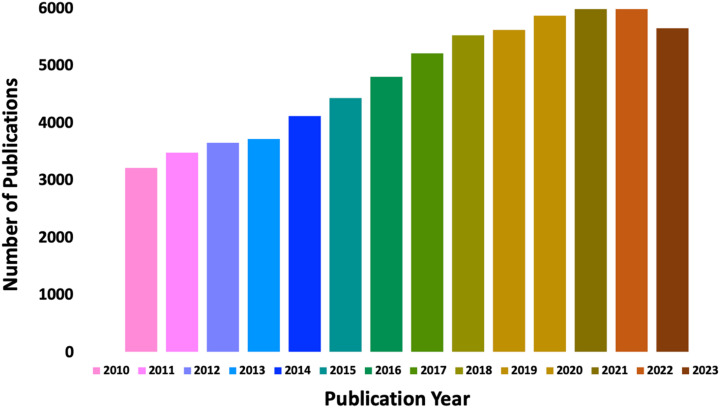
Schematic representation of the total number of papers published between 2010–2023 under polymer chemistry with light and photochemistry aspects.

**Scheme 1:**
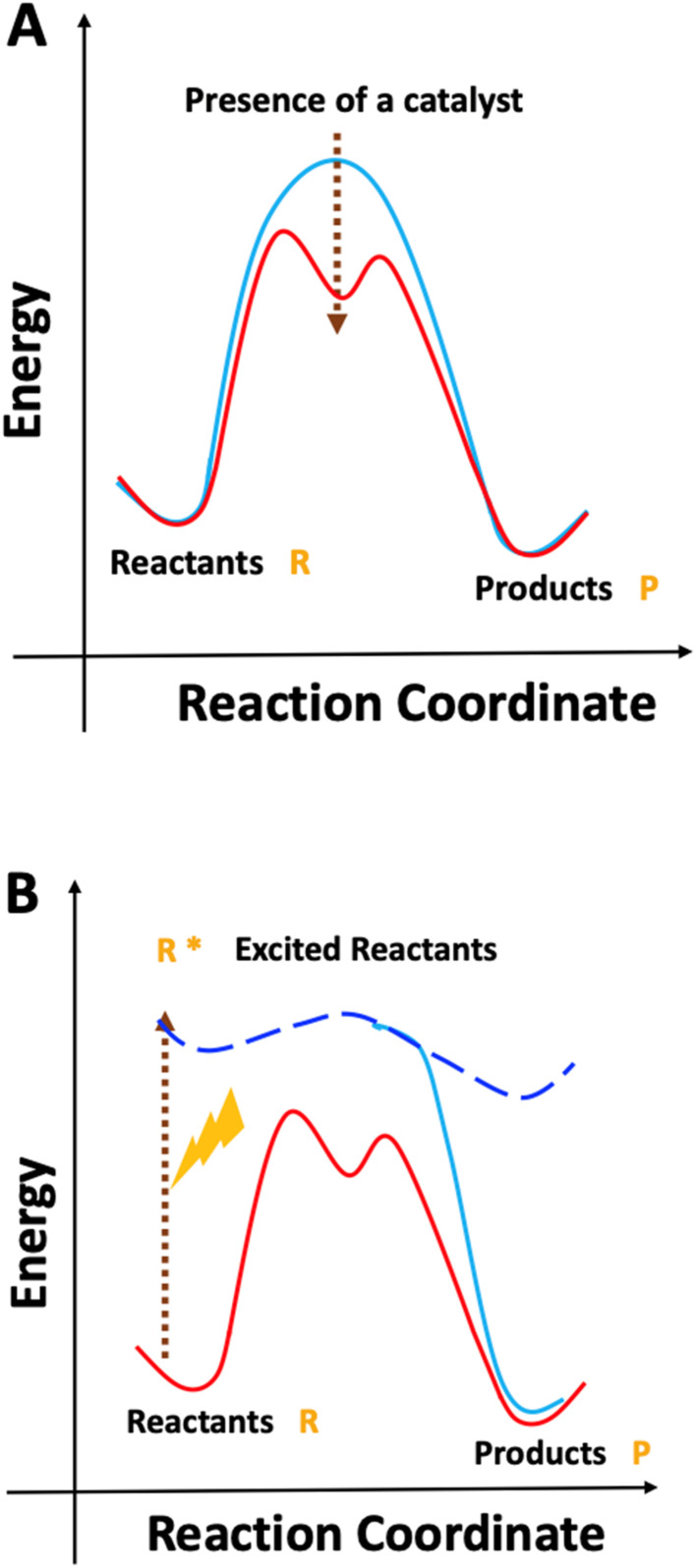
Reaction activation under (A) thermal and (B) photochemical conditions. Scheme inspired by Fig. 1 of ref. 7.

Synthetic polymer photochemistry is a growing area of modern polymer science. Since 2010 there has been a substantial growth in the number of papers that combine polymer chemistry with either light or photochemistry, as seen in Fig. 1. The appeal of photochemistry comes from its ability to impart a high amount of energy to molecules, thereby allowing the molecules to access reactions that are otherwise very challenging under thermal conditions. This is demonstrated schematically in Scheme 1.

Indeed, much of modern polymer photochemistry uses visible light with wavelengths in the range of 380–700 nm or UV radiation with wavelengths in the range of 200–380 nm.^8^ The energy (*E*) imparted to a molecule by absorbing a photon can be estimated using the Planck equation below:^9^1
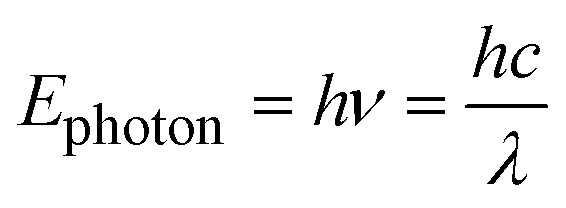
where *h* is the Planck constant (6.626 × 10^−34^ J s), *ν* is the photon frequency, *c* is the speed of light (3.00 × 10^8^ m s^−1^) and *λ* is the photon wavelength. In contrast kinetic energy from the thermal environment can be approximated by using the relationship below:^10^2
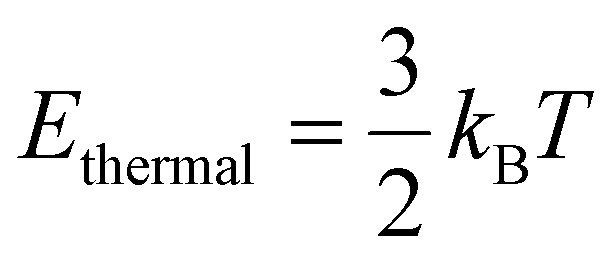
where *k*_B_ is the Boltzmann's constant (1.38 × 10^−23^ J K^−1^) and *T* is the absolute temperature. Eqn (2) is strictly true only for ideal gas molecules, however, it provides an estimate of the thermal energy available to molecules more broadly. Substituting typical values of a violet photon at the edge of visible and UV radiation of 400 nm and ambient temperature of 298 K gives *E*_photon_ ≈ 5 × 10^−19^ J, while *E*_thermal_ ≈ 6 × 10^−21^ J. Even considering some of the highest terrestrial temperatures such as the center of the earth at 6000 K (ref. 11) gives thermal energy in the order of *E*_photon_ ≈ 1 × 10^−19^ J. This highlights the potential of photochemistry, since easily accessible photons in the visible or near UV ranges have energies that exceed, by several orders of magnitude, the ambient thermal energy available under lab conditions, and even exceed thermal energy available in extreme terrestrial thermal locations. However, one note that needs to be made in this comparison is that thermal energy is available to all molecules in a reaction mixture. In contrast, the high energy of photons is only available to the one molecule that absorbs the photon, necessitating a continuous photon flux to drive photochemical reactions.

**Fig. 2:**
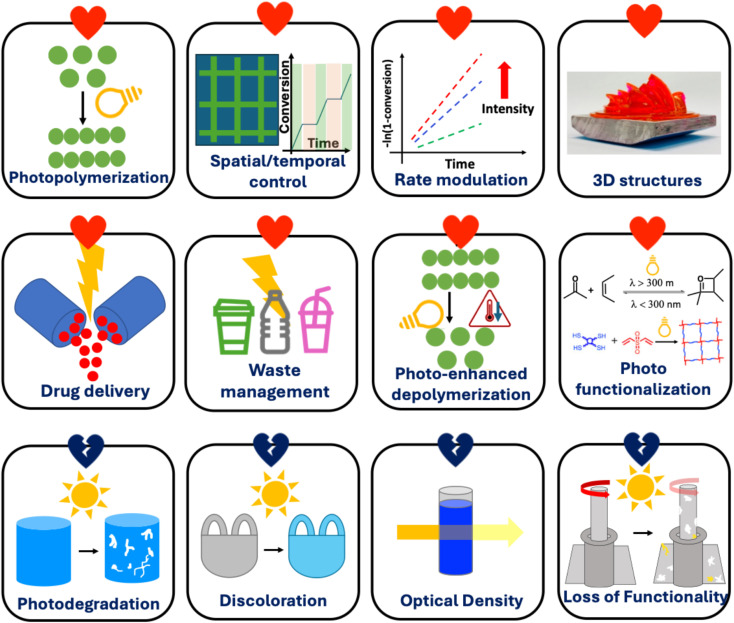
Interactions of light with polymers.^12–16^

The high energy of photons enables unique chemical reactions to occur in polymer systems. This can include the generation of polymers, their transformation to functional materials, and even their decay and degradation. Each of these three areas will be highlighted in this perspective. This perspective article is not a comprehensive summary of all possible transformations involving polymers under light but rather highlights unique possibilities in the generation, transformation, and disassembly/degradation of polymers under light. The contribution focuses on how polymers can grow or be enhanced by light, or how they can be degraded or be destroyed under light (Fig. 2). It is important to note that both the photophilic and photophobic nature of polymers offer unique opportunities in materials engineering, and this perspective aims to highlight both.

### Photopolymerization

Light driven polymerization has been known for many years; indeed, in the 1840s the light driven reactions of styrene under light were noted to form glass like products, far earlier than radical polymerization was understood or even macromolecules.^17,18^ This was understood to be a chain reaction in the early 1910s, showing that the amount of poly(vinylbromide) increased under light.^19^ Despite being established for well over a century in some form, light driven polymerization has taken off in the past decade in part due to new developments in controlled polymerization using highly reactive cationic and radical intermediates.^20^ Additionally, the recent development of 3D printing and additive manufacturing technologies has created an impetus for new methods of generating polymers under light, which can then be converted to macroscopic structures that have never been generated before.^21–23^

Industrially, photopolymerization is commonly used to create functional surfaces or for chlorination of compounds.^24^ The main benefit of photopolymerization is the ability to generate reactive species, at ambient or even sub-ambient temperatures. This has both energetic benefits, since heating can be expensive, and can also increase the yield of polymers with low ceiling temperatures, where polymerization under heat may be thermodynamically unfavorable.^25^

**Scheme 2:**
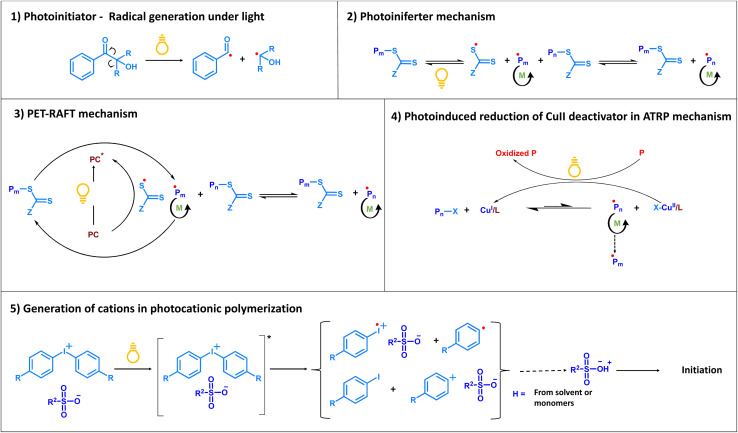
Reaction mechanisms for generation of reactive species under light. (1) Radical generation from photoinitiators.^26^ (2) Photoiniferter mechanism.^27,28^ (3) PET-RAFT mechanism.^28^ (4) Photoinduced reduction of the Cu^II^ deactivator complex in ATRP.^29^ (5) Generation of cations and strong acids in photocationic polymerization from cationic photoinitiators.^30^

Examples of reactive species generated by photochemical processes include radicals from classical photoinitiators,^26^ cleavage of the C–S bond through the photoiniferter process,^27,28^ photocatalytic generation of radicals by energy or electron transfer processes in photoinduced electron/energy transfer reversible addition fragmentation chain transfer polymerization (PET-RAFT),^28^ reduction of Cu^II^ deactivator complexes through amines in photochemical atom transfer radical polymerization (ATRP),^29^ generation of cations in photocationic polymerization, photoacid^30^ based photochemical activation of metathesis catalysts, and even activation of monomers in ring opening metathesis polymerization (ROMP)^31^ in the presence of appropriate mediators. These are mechanistically depicted in Scheme 2.

After the initial discovery of RAFT polymerization, it has become one of the most powerful techniques for synthesizing polymers with excellent control and living characteristics.^32^ Since then, various aspects of RAFT polymerization have been developed. For instance, although thermal activation was applied to initiate the traditional RAFT process, later photoinitiation was introduced to perform a reaction at room temperature. Mainly, three techniques were coupled with RAFT polymerization including using light to dissociate a photo initiator (photo-RAFT)^33^ or using light to directly dissociate the thiocarbonylthio compound in the absence of a photoinitiator (photoiniferter polymerization)^34^ or using light to excite a photocatalyst to initiate the polymerization (PET-RAFT polymerization).^35^ Ostu and coworkers polymerized methyl methacrylate (MMA) in the presence of UV irradiation in 1984.^36^ Since then, UV activation for the dissociation of a photoinitiator or chain transfer agents (CTA) has been used in both research and industrial applications. In most cases, UV activation has some potential issues with decomposition of CTA, thereby resulting in polymers with poor control and non-living characteristics.^37,38^ However, UV activation is used in various industrial manufacturing processes where radical polymerization is applied to synthesize polymers and in experiments where CTA deprotection is needed. Even thoughUV is used in various industrial radical polymerization processes such as paints and coatings,^39,40^ dentistry and photo curing 3D printing,^41^ low penetration of UV is always challenging.^42,43^ Lalvée and coworkers reported applying near-infrared (NIR) light in methacrylate polymerization.^43^ NIR has low energy compared to UV, although NIR is a more desirable choice because of NIR's ability to penetrate deeper into materials in photocuring applications and non-toxicity in biological applications. Therefore, a three-component system including a borate dye, phosphine, and iodonium salts were used in the study to achieve higher conversion under NIR irradiation. In the process, the borate dye acts a photosensitizer and iodonium salt acts as the photoinitiator, while phosphine is the key component to prevent polymerization inhibition by oxygen and to regenerate the dye.

In 2014, Boyer and coworkers developed PET-RAFT polymerization to perform a RAFT-polymerization *via* photochemically generated radicals under visible light with excellent control and living characteristics.^44,45^ In that study, they elegantly showed the capability of PET-RAFT polymerization to synthesize polymers with excellent control starting from both conjugated and unconjugated monomers in the presence of the *fac*-tris[2-phenylpyridinato-C_2_,*N*]iridium(iii) (Ir(ppy) _3_) photocatalyst under blue light irradiation. Light excites the photocatalyst to an excited state which then interacts with a CTA to generate initiating species in the PET-RAFT mechanism since the CTA acts as both an initiating and transfer agent.^35,45^ The selection of a suitable photocatalyst depends on several parameters mainly including monomer compatibility, solubility, and ability to result in high monomer conversion and better control. Therefore, having a universal photocatalyst with superior qualities like higher photostability, a broad absorption spectrum, solubility, and compatibility in any photo polymerization technique would be highly desirable and ideal. However, this area is yet to be investigated further.

Recently, Konkolewicz and coworkers showed the importance of the PET-RAFT process in the uniformity of a network compared to conventional photo-RAFT polymerization.^46^ The impact of the photocatalyst in the PET-RAFT process on the network's uniformity was investigated.^46^ Ir(ppy)_3_ and zinc tetraphenylporphyrin (ZnTPP) were used in the PET-RAFT process and phenylbis(2,4,6-trimethylbenzoyl) phosphine oxide (BAPO) in the photo-RAFT process were compared with respect to the synthesis of a uniform crosslinked methyl acrylate (MA) network. Lower dispersity polymers in gel permeation chromatography (gpc) analysis and higher swelling ratios were found in the ZnTPP system compared to both Ir(ppy)_3_ and BAPO systems. Higher swelling ratios derive from more uniform networks because of their ability to expand properly and to hold more solvent compared to networks with irregular crosslinker distributions. It has been stated that ZnTPP undergoes oxidative electron transfer and Ir(ppy)_3_ undergoes energy transfer in the PET-RAFT mechanism.^47,48^ Therefore, the more uniform polymers made in the ZnTPP PET-RAFT process are proposed to occur through the efficient radical deactivation through the more prominent reversible reaction of the propagating radical with the CTA group under diffusion control in viscous networks, in addition to RAFT exchange. While poorer control in the BAPO intiated photo-RAFT mechanism occurs because that system can only have radical deactivation by RAFT exchange, which is less efficient in viscous polymer networks. This example demonstrated the importance of the choice of the photocatalyst in the PET-RAFT mechanism.

Furthermore, despite all the impressive advantages of photo polymerization, the presence of a residual photocatalyst in the final system is one of the major drawbacks which can limit its potential to be used in different applications and can lead to potential polymer degradation or undesirable side reactions. Although early studies used mostly transition metal containing photocatalysts such as Ir(ppy)_3_, ZnTPP or tris(bipyridine) ruthenium(ii) chloride (Ru(bpy)_3_Cl_2_), the presence of even a small amount of a metal catalyst could be an issue for biological applications and can cause negative impacts on the environment.^49,50^ Therefore, various metal free nontoxic organic photocatalysts such as Eosin Y, methylene blue, Nile red, fluorescein, rhodamine 6G, and oxygen-doped anthanthrene have been applied and have led to an efficient PET-RAFT process with irradiation in the visible regime.^51,52^ Similar to the PET-RAFT process, other photopolymerization techniques more desirably use metal free photocatalysts. For instance, phenoxazine, phenothiazine, dihydrophenazine, and oxygen-doped anthanthrene are some examples of metal free photocatalysts utilized in photo-ATRP.^53^

In addition to this, other alternative techniques were introduced such as using amine in a catalyst free system. Boyer and coworkers synthesized a heterogeneous Eosin Y-silica nanoparticle conjugated system (EY-SNP) with the ability to be reused, better photodegradation tolerance and the ability to achieve high monomer conversion for both hydrophilic and hydrophobic monomers in the presence of low catalyst concentrations (3–7.5 ppm).^54^ Covalent conjugation of Eosin Y with silica nanoparticles has increased stability of the system under light, in contrast to free Eosin Y. Additionally, the silica-Eosin Y nanoparticles improve the efficiency of the catalyst removal process by simple centrifugation. Furthermore, the reusability was tested for up to 5 cycles with monomer conversion higher than 70% at each cycle and, experimental *M*_n_ well agrees with theoretical *M*_n_ and low dispersities. Although the initial dark orange color of the EY-SNP system was consistent after 12 h polymerization time, the initial pale orange color of free Eosin Y changed to yellow showing the eventual loss of the photocatalyst.

Although efficient photopolymerization has been extensively used at the laboratory scale, challenges persist, including the high cost of photocatalysts (mitigated partially by ppm loadings) and the challenges associated with separating the catalyst from the polymer, especially in the case of polymer networks.

### Mild polymerization conditions

In most efficient polymerization methods, the dominant reaction is propagation; without this high molecular weight polymers could not be generated. However, competing with the desirable propagation steps are undesirable side reactions such as chain ending irreversible termination and transfer events. In many cases these undesirable reactions have higher activation energies than the desired propagation steps. In these cases, the fraction of chains affected by irreversible transfer or termination can be suppressed by using ambient or sub-ambient temperatures. However, in chain growth processes reactive sites must be generated. In a thermal process the generation of reactive cations or radicals strongly depends on temperature. For instance, in radical polymerization, at room temperature the most commonly used radical initiator, azobisisobutyronitrile (AIBN) has a half-life of approximately 200 days at 25 °C.^55^ Even the lowest temperature commercially available initiator, 2,2′-azobis(2,4-dimethyl-4-methoxyvaleronitrile) (V-70), has a half-life of approximately 20 h at 25 °C and a half-life of 44 days at 0 °C.^55^ This makes radical chemistry at room temperature challenging, and virtually impossible below room temperature using thermal initiation. Low temperature polymerizations are highly important for biologically relevant reactions where high temperatures may damage the biological species of interest.

**Fig. 3:**
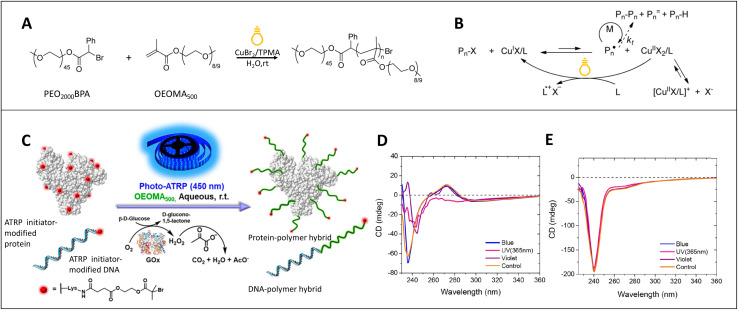
(A) Photoinduced ATRP with a Cu catalyst (ppm-level) under visible light irradiation in aqueous media. (B) Reaction mechanism of photoinduced ATRP in aqueous media. Reaction conditions: [OEOMA_500_]_0_/[PEO_2000_BPA]_0_/[CuBr_2_]_0_/[TPMA]_0_ = 450/1/*x*/4*x* in 90% (v/v) water with 392 nm irradiation at 0.9 mW cm^−2^ at room temperature (reproduced from ref. 59 with permission from American Chemical Society, copyright 2015).^59^ (C) Synthesis of polymer bioconjugates *via* photoinduced ATRP under blue light irradiation. Reaction conditions: the ATRP initiators were modified by linking the protein (BSA) surface or DNA to the initiator. The polymerization was performed under blue light 450 nm irradiation and by applying a glucose (Glu), Gox, and sodium pyruvate (SP) deoxygenating system to achieve more biocompatible oxygen removal compared to N_2_ bubbling. (D) CD measurements of GOx under different light irradiations at room temperature for 2 h. (E) CD measurements of BSA under different light irradiations at room temperature for 2 h (adapted from ref. 60 with permission from American Chemical Society, copyright 2018).^60^

As highlighted in the Introduction the high energy of photons from visible or UV light sources greatly surpass the thermal energy available. This makes purely photochemical processes essentially temperature independent.^56–58^ Therefore, generation of reactive species such as radicals, anions or active catalysts can be achieved photochemically either under ambient conditions or even at lower temperatures. Two elegant examples of photochemical polymerization under ambient conditions are shown in Fig. 3. Pan *et al.* performed photoinduced ATRP in an aqueous medium with a ppm level Cu catalyst under visible light irradiation at 0.9 mW cm^−2^ with 392 nm showing its ability to prepare well controlled polymers with living characteristics in an aqueous medium.^59^ The polymerization reaction and the photoinduced ATRP equilibrium in aqueous media are illustrated in Fig. 3A and B. This reaction shows the ability of photoinduced ATRP to be used in various applications including biological applications because of mild reaction conditions such as low catalyst concentration, aqueous medium, visible light irradiation, and room temperature. Summerlin and coworkers synthesized protein–polymer conjugates through PET-RAFT polymerization with blue light irradiation.^61^ Blue light is often recognized as an ideal candidate in biological applications as an energy source compared to UV, because UV can denature a protein's structure or cause mutations to DNA. Furthermore, excess purifications are mostly vital in applying traditional ATRP methods (ICAR ATRP or ARGET) for synthesizing bioconjugates because of introducing external components such as initiators, reducing agents or higher concentrations of ligands to the system compared to photo-ATRP with relatively minimum purifications.^60^ As seen in Fig. 3C, Fu *et al.* applied photoinduced ATRP in an aqueous medium to synthesize protein/DNA-polymer conjugates following the “grafting-from” method.^60^ Interestingly, they showed the impact of the energy source on the protein's structure by performing circular dichroism (CD) experiments for glucose oxidase (GOx) and bovine serum albumin (BSA) proteins and found that blue light is capable of preserving the native structure of GOx and BSA as shown in Fig. 3D and E respectively.

In addition to applying light to achieve mild conditions in polymer synthesis, recently, light was combined with thermal energy to accelerate polymer depolymerization and to perform depolymerization under mild conditions.^16^ This concept provides a significant temperature reduction for depolymerization under photothermal conditions compared to thermal conditions and is discussed later under photo-enhanced depolymerization.

**Fig. 4:**
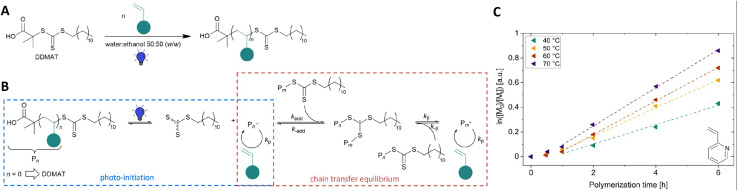
(A) Synthesis of macroCTAs *via* blue light-induced iniferter RAFT polymerization under blue light irradiation. (B) Mechanism of the blue light-induced RAFT polymerization in the presence of the DDMAT (2-(dodecylthiocarbonothioylthio)-2-methylpropionic acid) RAFT agent. (C) Pseudo first-order kinetic plots of 2VP polymerization on the 40 mL scale at temperatures of 40–70 °C under blue light irradiation ([2VP]_0_/[DDMAT]_0_ = 470, 19.7 V) (reproduced from ref. 62 with permission from Royal Society of Chemistry, copyright 2023).^62^

One important factor to note for performing polymerization under light is that polymerization includes a temperature independent process, which is the photochemical generation of reactive species, which then undergo thermal processes such as propagation. These thermally driven propagation steps lead to photopolymerization still having a temperature dependence, even though they are based on photochemical initiation processes. Abetz and coworkers explored the impact of reactor temperature on photoiniferter RAFT polymerization of 2-vinylpyridine (2VP) and found a significant temperature dependent behavior as illustrated in Fig. 4C. Notably, a higher apparent propagation rate (*k*_p_,_app(70°C)_ = 0.155 h^−1^) was achieved at 70 °C compared to *k*_p_,_app(40°C)_ = 0.085 h^−1^ at 40 °C due to the presence of more thermal energy at high temperature to generate reactants with energy higher than the activation energy of monomer addition.^62^ Recent work has further highlighted the impact of temperature on the apparent activation energy for photoinitiated radical copolymerization both under conventional and RAFT conditions.^63^ In most cases, the known activation energies of propagation and termination can be combined to provide good agreement with the observed impact of temperature on the photopolymerization rate. However, RAFT photopolymerization using a somewhat or strongly retarded monomer leads to a particularly strong temperature dependency. This is both a strength, as it allows temperature to be used as a handle to tune reaction rates, but also a limitation as it can lead to reductions in the efficiency of photopolymerization.

### Modulation of rates

Photochemical radical polymerizations involve a steady state concentration of radicals, determined from the photochemical radical generation processes and radical loss due to termination processes. Therefore, the rate of the reaction is intimately tied to the light intensity. A body of research has found that the rate of radical photopolymerization under both conventional free radical polymerization (FRP) and RAFT conditions such as PET-RAFT even using intrinsic monomer initiation leads to a scaling of the polymerization rate with light intensity as:^64^3
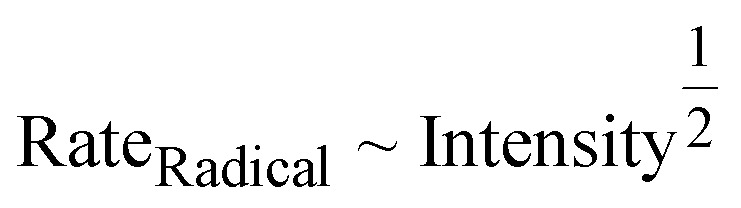


**Fig. 5:**
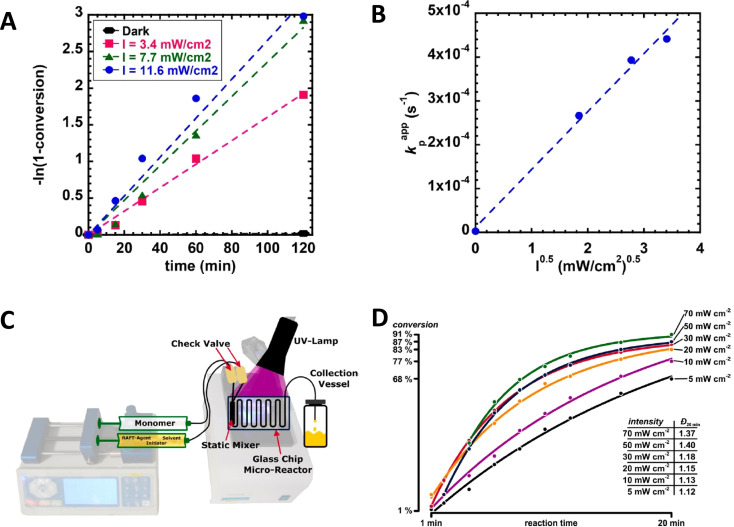
(A) Semilogarithmic kinetic plot of PET-RAFT polymerization of MA at different blue light intensities (440 ± 10 nm). (B) Scaling relationship of the apparent propagation rate (*k*_p,app_) with the reciprocal square root of photoreactor intensity (*I*^0.5^). Reaction conditions for A and B graphs = [MA]_0_ : [PADTC]_0_ : [Ir(ppy)_3_]_0_ = 200 : 1 : 0.001, [MA]_0_ = 4.9 M, in DMSO : DMF = 7 : 3, 5.9 mL total, at ambient temperature (PADTC-2-propanoic acid dodecyltrithiocarbonate). Reactor intensities were 11.6 ± 0.3, 7.7 ± 0.3, and 3.4 ± 0.3 mW cm^−2^ (reproduced from ref. 64 with permission from American Chemical Society, copyright 2018).^64^ (C) Sketch of a microflow reactor for photo-RAFT polymerization. (D) Impact of different light intensities on the reaction rate of a photoinitiated RAFT polymerization of *n*-butyl acrylate (*n*BA) in a microflow reactor. Conditions for C and D figures: light source = Omnicure S1000 system (wavelength = 365 nm). Ratios of (DoPAT) : benzoin : *n*BA = 1 : 0.25 : 80 were used in the presence of *n*-butyl acetate (BuAc) solvent (DoPAT-2-(dodecylthiocarbonothioylthio)propanoic acid). All reactions were performed at 60 °C (reproduced from ref. 65 with permission from American Chemical Society, copyright 2016).^65^

**Scheme 3:**
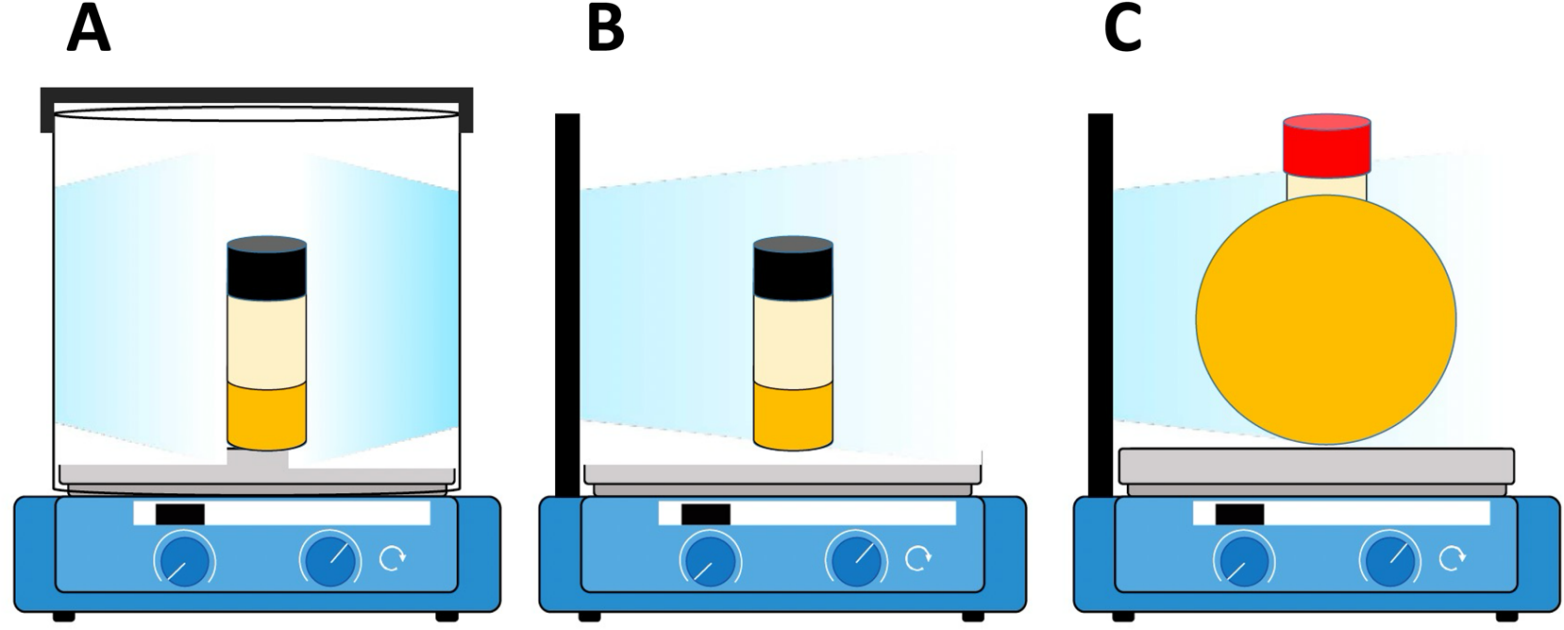
Schematic representation of different light sources/photoreactors. (A) Cylindrical light source/photoreactor with a cylindrical reaction vessel setup. (B) Flat light source/photoreactor with a cylindrical reaction vessel setup. (C) Flat light source/photoreactor with a spherical reaction vessel setup. All set ups including a reaction vessel filled with the reaction mixture and oil were placed inside an aluminum foil-covered photoreactor on a stir plate (reproduced from ref. 66 with permission from Elsevier, copyright 2023).^66^

Indeed, the rate of polymerization can be carefully modulated using these principles. Examples of controlled photochemical radical, RAFT based reactions demonstrating this ½ order of polymerization rate on light intensity are given in Fig. 5A and B.^64^ Beyond light intensity, the geometry of the reaction setup can have a significant impact on the outcome and rate of polymerization.^66^ Commonly used batch geometries are shown in Scheme 3. Flow systems reduce many of these limitations, by effectively increasing the available surface area that can be irradiated in well-designed systems, although this still may remain. In a study, Junkers and coworkers explored the importance of several factors including the ratio of the photoinitiator, temperature and light intensity to achieve a better polymerization rate and good control in continuous flow photo-RAFT polymerization as depicted in Fig. 5C. The change in polymerization rates with the light intensity is shown in Fig. 5D. The reaction rate increases on increasing the light intensity staring from 5 mW cm^−2^ to 30 mW cm^−2^. However, there was a negligible difference in the polymerization rate in the presence of intensities higher than 30 mW cm^−2^ because of the full light saturation of the photo reactor.^65^

Similarly, cationic photopolymerizations are also intensity dependent, but since cations are not able to self-terminate, a simpler relationship is observed. In these cases, for cationic polymerizations the rate of reaction scales as a linear function of light intensity as seen below:^67^

4 Rate_Cationic_ ∼ Intensity

**Fig. 6:**
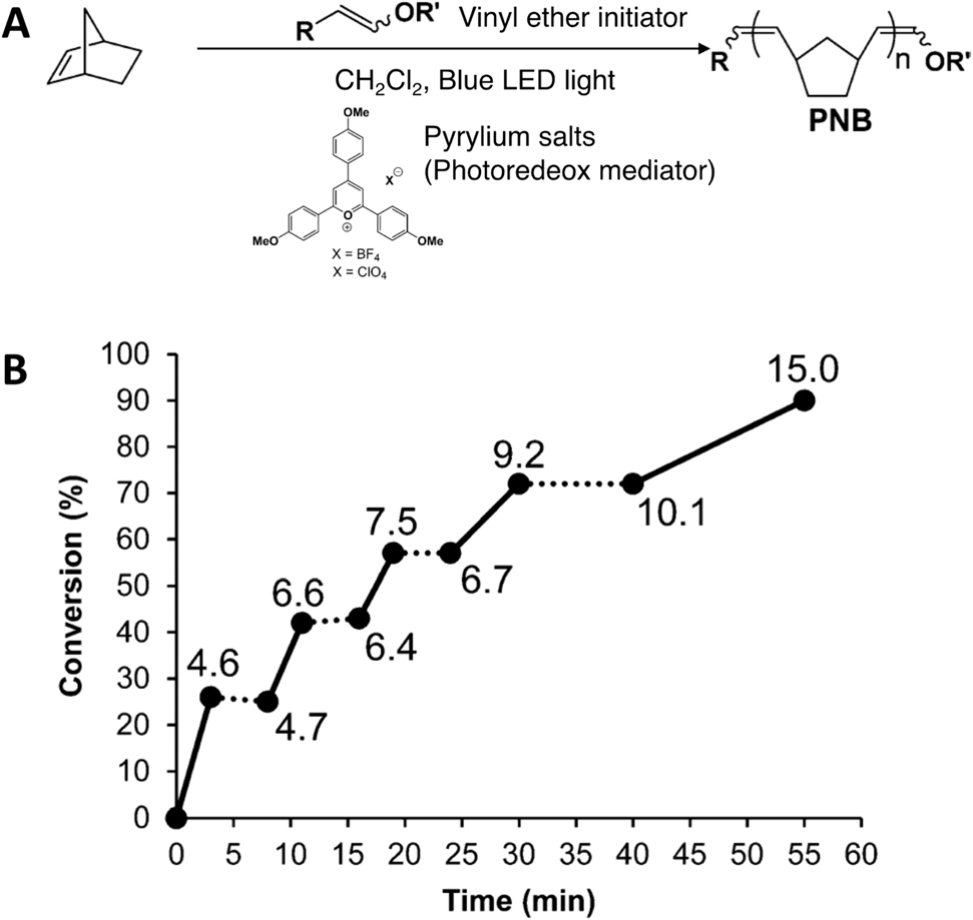
(A) Photo redox mediated ROMP. (B) Plot of % conversion of the monomer *vs.* time. Solid lines represent periods of exposure to blue light. Dashed lines represent periods in the dark. *M*_n_ values (kDa) are shown at each point. Initial conditions: monomer : vinyl ether initiator = 100 : 1, [monomer]_0_ = 1.9 M (adapted from ref. 68 published Open Access by American Chemical Society, copyright 2015. This is an unofficial adaptation of an article that appeared in an ACS publication. ACS has not endorsed the content of this adaptation or the context of its use).^68^

Many photochemically triggered ROMP reactions involve the transformation of a latent catalyst to an active form.^30^ This leads to a light activated initiation but once an active metathesis catalyst is generated it will continue to consume monomers until depletion. This leads to a much weaker dependence of polymerization rate on light intensity since light is not used to continuously drive polymerization by replacing lost reactive species. However, as shown in the ROMP reaction in Fig. 6, conversion was obtained in the presence of blue light, with no conversion and recontinuation of the reaction in the dark and under light reirradiation, respectively. This was the first example with metal free ROMP initiated with one-electron oxidation of vinyl ether initiators *via* electrochemical or photo redox approaches. A better polymerization in shorter reaction time has been achieved in a photo redox process. In the proposed mechanism, polymerization is initiated by reacting a cycloalkene monomer with a vinyl ether radical cation which resulted *via* the photo redox mediated one-electron transfer mechanism facilitated by a pyrylium photo redox mediator. The propagating chain bears a radical cation at the end which reacts with monomers to continue the polymerization. This propagating chain with a radical cation end and the reduced pyrylium version act as a dynamic redox couple which has the ability to end polymerization, reducing the radical cation and oxidizing the reduced version of the pyrylium photo redox mediator. Upon reirradiation, polymerization reoccurs. Based on the correlation of monomer conversion with increasing *M*_n_, Boydston and coworkers suggested the occurrence of chain end activation–deactivation cycles rather than the formation of new polymers *via* a photo mediated process under reirradiation of light. Furthermore, a longer polymerization time was required in the presence of light irradiation by half of the bulbs.^68^

However, coupled with the dependence of the photopolymerization rate on light intensity is the inevitable challenge of optical density.^69^ Optical density arises from the chromophores closer to the light source absorbing photons, making them unavailable for molecules further away from the light source. This greatly reduces the ability to modulate the rate using light intensity, especially in cases where samples are optically dense. Although inherent to photochemistry, several approaches have been attempted to mitigate or reduce the impact of optical density on photopolymerization. The most commonly used approach is to increase the available reaction vessel surface area using flow type systems.^70^

An alternative approach was proposed, by combining light generating reactions through chemiluminescence and photochemical radical initiation in discrete reaction phases, specifically having photopolymerization in an aqueous phase with chemiluminescence occurring in an intermixed but discrete organic phase.^71^ Nevertheless, optical density remains an unresolved challenge in the field of polymer photochemistry which results from the very nature of the delivery of photons to photochemical reactions.

### Temporal/spatial control

**Fig. 7:**
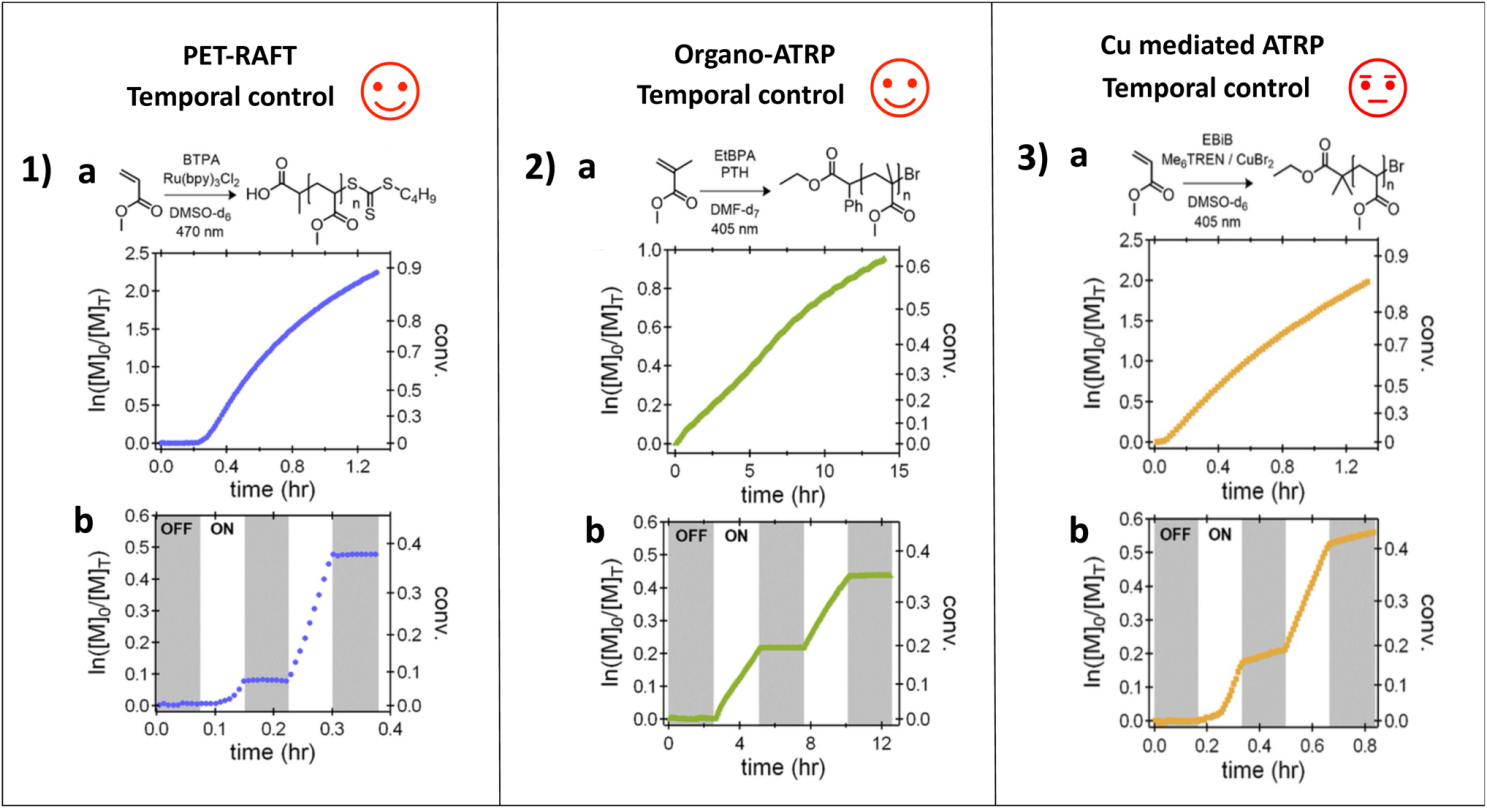
(1) PET-RAFT polymerization of MA using 470 nm light irradiation in the presence of tris(2,20-bipyridyl)dichlororuthenium(ii) hexahydrate ([Ru(bpy)_3_]Cl_2_·6H_2_O). (a) Kinetic plots of the polymerizations at a fixed photon flux. (b) Temporal control experiments for the PET-RAFT polymerization demonstrate ideal temporal control under PET-RAFT conditions. (2) Cu-free ATRP polymerization of MMA using 405 nm light irradiation in the presence of 10-phenylphenothiazine (PTH). (a) Kinetic plots of the polymerizations at a fixed photon flux. (b) Temporal control experiments for Cu-free ATRP polymerization demonstrate ideal temporal control under Cu-free ATRP conditions. (3) Cu-mediated RDRP polymerization of MA and MMA using CuBr_2_ and tris[2-(dimethylamino)ethyl] amine (Me_6_TREN). (a) Kinetic plots of the polymerizations at a fixed photon flux. (b) Temporal control experiments wherein distinct linear growth during dark periods after initial irradiation is observed for both polymerizations (∼10–15% of the “on” rate) (adapted from ref. 76 with permission from John Wiley and Sons, copyright 2019).^76^

As identified above, the photopolymerization rate can be modulated by the light intensity. The extreme case of intensity control over polymerization is the phenomenon that in the absence of light, photopolymerization should not occur. This induces temporal control over photopolymerization. A hallmark of this temporal control is the classic “on–off” type experiments where polymerization occurs efficiently in the presence of light but shuts down in the absence of light. This has been demonstrated extensively in radical^13,72^ and cationic photopolymerization.^73–75^ This is because in the absence of photochemical driving forces the radicals and cationic species present at low concentrations simply combine or deactivate quickly removing reactive chain ends from the system.^76^ Photochemical ATRP presents a particularly interesting example of this phenomenon, since in photo-ATRP the active species generated is the Cu^I^ activator complex, which can be present at concentrations in the μM or even mM range, substantially higher than typical radical concentrations. These Cu^I^ species can persist well into the dark period, generating radicals that eventually terminate. In a comparative study, Hawker *et al.* showed that the nature of the photocatalytic system has a marked effect on the sharpness of rate transitions from the light to dark period. Systems using photocatalyst excitation, such as PET-RAFT or organoATRP showed very sharp transitions between the light and dark states, while the presence of the Cu^I^ species that can extend into the dark period reduces the sharpness of the transition between light and dark periods.^76^ In a follow up study, Matyjaszeski *et al.* found that the sharpness of transitions can be improved by increasing the ATRP catalyst activity, since this allows the same rate of reaction to occur at lower concentrations of Cu^I^, implying that fewer radical termination events are required to lead to the complete conversion of all Cu species to Cu^II^ deactivators.^77^ These temporal control reactions are highlighted in Fig. 7.

In ROMP systems, temporal control has been also demonstrated, for example by the activation of monomers in metal free ROMP. This relies on the well-established photocatalyst principles, where only the excited state catalyst can activate the mediator through an electron transfer mechanism.^68,78^ Due to the possibility of back reduction of the alkenes by the photocatalyst, continuous light irradiation is needed to continue polymerization. In the absence of light, even if temporary, the polymerization ceased almost immediately.

**Fig. 8:**
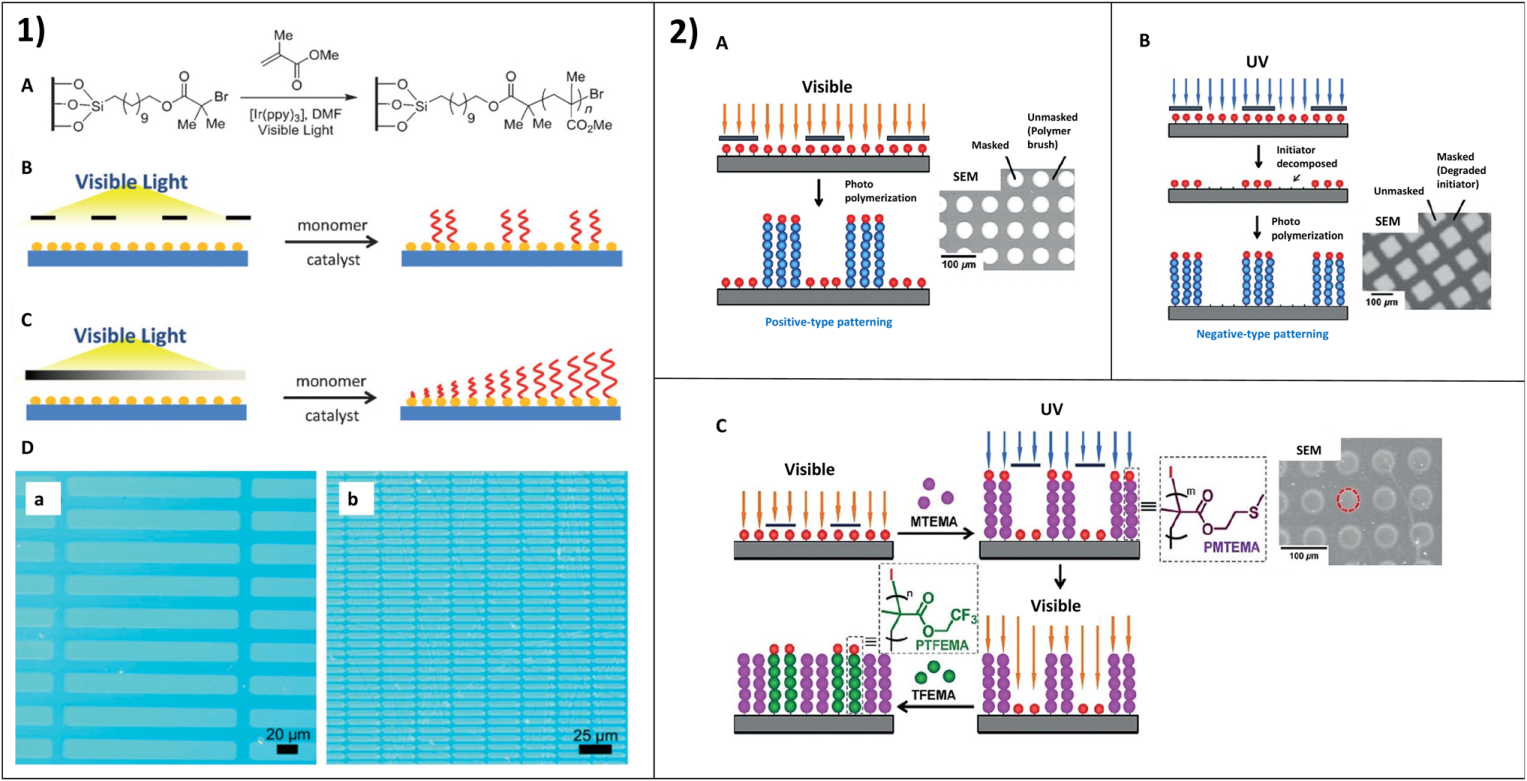
(1) (A) Patterning of polymer brushes from substrates uniformly functionalized with trichlorosilane-substituted bromoisobutyrate based initiators. (B) Creating patterns using a photomask. (C) Creating gradient structures using a neutral density filter. (D) Optical microscopy images of patterned PMMA brushes obtained using a negative photomask with (a) 20 mm by 200 mm and (b) 2.5 mm by 25 mm rectangles (reproduced from ref. 12 with permission from John Wiley and Sons, copyright 2013).^12^ (2) (A) Positive patterning of poly(benzylmethylacrylate) (PBzMA) brushes and a SEM image of the pattern. (B) Negative patterning of PBzMA brushes and a SEM image of the pattern. (C) Synthesis of a patterned binary polymer brush and a SEM image of the pattern. TFEMA = fluorinated monomer-2,2,2-trifluoroethyl methacrylate, and MTEMA = sulfur-containing monomer-2-(methyl methylthio)ethyl methacrylate (reproduced from ref. 79 with permission from John Wiley and Sons, copyright 2018).^79^

Coupled with the ability to control when polymerization occurs by turning light off or on, the same principle allows polymerization to be restricted to specific spatial regions. This is significant since it allows spatial control. Elegant examples of this have generated spatially resolved surface patterns where photopolymerization generated surfaces of polymers where irradiation occurs, while the other regions have essentially no polymer formed. Hawker and coworkers fabricated complex 3D-surface patterning by growing polymer brushes on a uniform layer of initiators (Fig. 8(1A)) with excellent temporal and spatial control through a visible light mediated radical polymerization. Interestingly, complex patterning structures or gradient structures were created using a photomask or a neutral density filter as illustrated in Fig. 8(1B and 1C). Optical microscopy images of patterns made by poly(methyl methacrylate) (PMMA) brush polymers show homogeneity in the brush growth with rectangular masking (Fig. 8(1D)) and this clearly demonstrates excellent spatial control over brush formation.^12^ Furthermore, in a study by Goto and coworkers, they prepared complex patterns by polymerizing different types of brush polymers including block polymers and binary polymers (two different brush polymers attached on to a surface) through photocontrolled surface initiated reversible complexation mediated polymerization (photo-SI-RCMP). As shown in Fig. 8(2), positive and negative-type patterns were achieved by masking some areas to block polymerization under visible light and decomposing the initiator tethered to some areas under UV light respectively. Specifically, the selectivity between visible and UV light has led to the formation of binary brush polymers and di-block polymers and thereby patterns.^79^

**Fig. 9:**
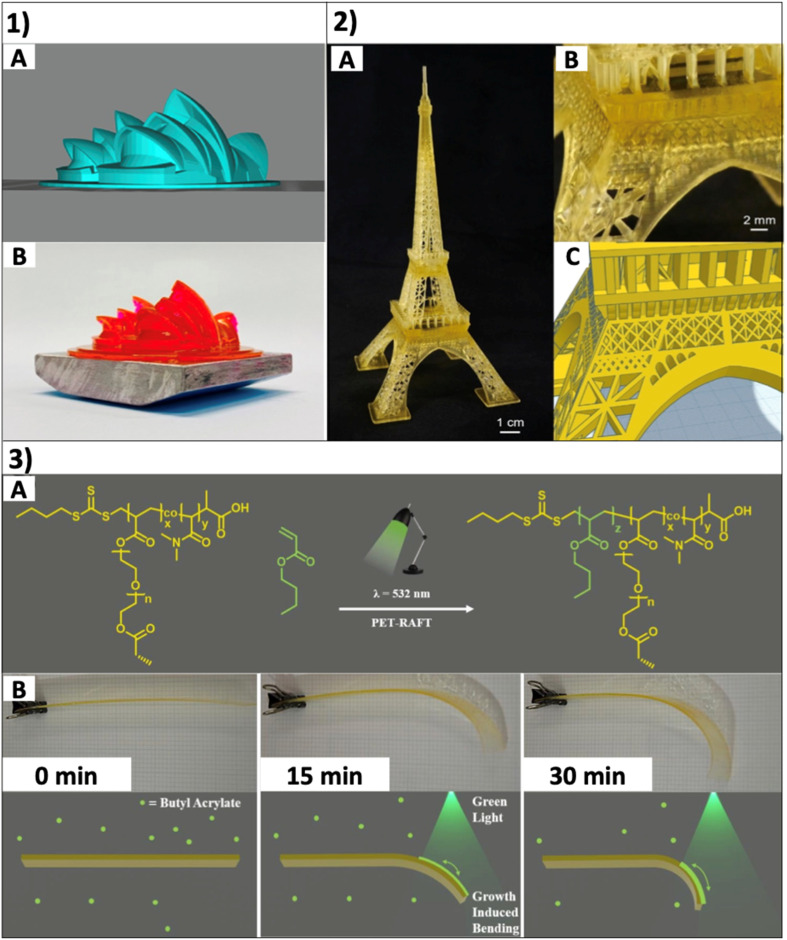
(1) (A) The original 3D model design of the theater complex. (B) Printed theater complex using the resin formulation of [PEGA]/[PEGDA]/[RAFT agent]/[EB]/[TEtOHA] = 571 : 1333 : 5:0.2 : 40. (PEGA-poly(ethylene glycol) methyl ether acrylate, EB-erythrosin B, and TEtOHA-triethanolamine) (reproduced from ref. 14 with permission from American Chemical Society, copyright 2021).^14^ (2) (A) Printed Eiffel Tower using novel resins containing BTPA as the RAFT agent. (A) Full-sized 3D printed object. (B) Close-up of a 3D printed figure showing details. (C) The original 3D model design. The object was printed using a ratio of [BTPA] : [PEGDA] : [DMAm] : [TPO] = 1 : 40 : 160 : 2 under violet light (*I*_max_ = 405 nm, *I*_0_ = 0.81 mW cm^−2^) irradiation. (BTPA-2-(butylthiocarbonothioylthio) propanoic acid) (reproduced from ref. 82 with permission from John Wiley and Sons, copyright 2021).^82^ (3) (A) PET-RAFT polymerization reaction of BA. (B) Optical and graphical images of the growth induced bending process. 0 min = the initial 3D-RAFT printed strip. 15 min = the 3D-RAFT strip after 15 minutes under monodirectional green light irradiation (532 nm, 58.72 μW cm^−2^) in a growth medium of DMSO and BA. 30 min = the same 3D-RAFT strip after 30 minutes under monodirectional green light irradiation in the same growth medium (reproduced from ref. 83 with permission from Royal Society of Chemistry, copyright 2020).^83^

Taking advantage of a similar principle, coupled with a moving stage allows the 3D printing of soft materials with complex geometric structures. Elegant examples of photochemical patterning have been realized on surfaces using for instance ATRP type approaches with subsequent advances to simplify the process and make it more accessible through metal free approaches.^80,81^ The types of surface patterns possible are shown in Fig. 9. 3D printing goes beyond simple control over where polymerization occurs on a 2D surface through light irradiation. Recent work has highlighted the potential of light driven methods to generate complex structures in 3D. The utilization of reversible deactivation radical polymerization (RDRP) methods such as PET-RAFT, photo-ATRP, photo-RAFT methods or photoinduced radical-promoted cationic RAFT is allows the synthesis of complex 3D structures with a homogeneous network, compared to traditional conventional radical photo polymerization which results in a heterogenous network. In addition to this, the presence of active end groups in the polymers from RDRP techniques can be used to introduce post printing modification.^84^ Boyer and coworkers demonstrated the ability of PET-RAFT polymerization and Norrish type I photoinitiated RAFT polymerization to synthesize complex 3D structures under mild visible light conditions.^14,84^ The 3D structures from PET-RAFT polymerization and Norrish type I photoinitiated RAFT polymerization are shown in Fig. 9(1) and (2) respectively. An elegant example of coupling 3D printing with spatial control of light to achieve 4D structures was reported by Jianyong Jin and coworkers. As seen in Fig. 9(3A), the printed polymer strip (copolymer of poly(ethylene glycol)diacrylate (PEGDA) and *N*,*N*-dimethylacrylamide (DMAm)) with living characteristics was chain extended in the presence of the butyl acrylate (BA) monomer under green light irradiation. Interestingly, as shown in Fig. 9(3B), a growth induced bending has been achieved because of spatial control with bending only in the light irradiated region compared to the region in the dark.^83^

Despite significant progress with regards to spatiotemporal control, there remain challenges. Since these processes can involve light irradiation followed by diffusion of the photogenerated reactive species such as radicals or excited state catalysts, this can reduce the fidelity of a pattern especially at small feature sizes. As highlighted earlier, temporal control requires essentially immediate deactivation of the active species and activator catalysts in the absence of light. This is not always universally possible, for instance as seen in the photo-ATRP examples.

### Photofunctionalization

#### Cycloadditions

**Scheme 4:**
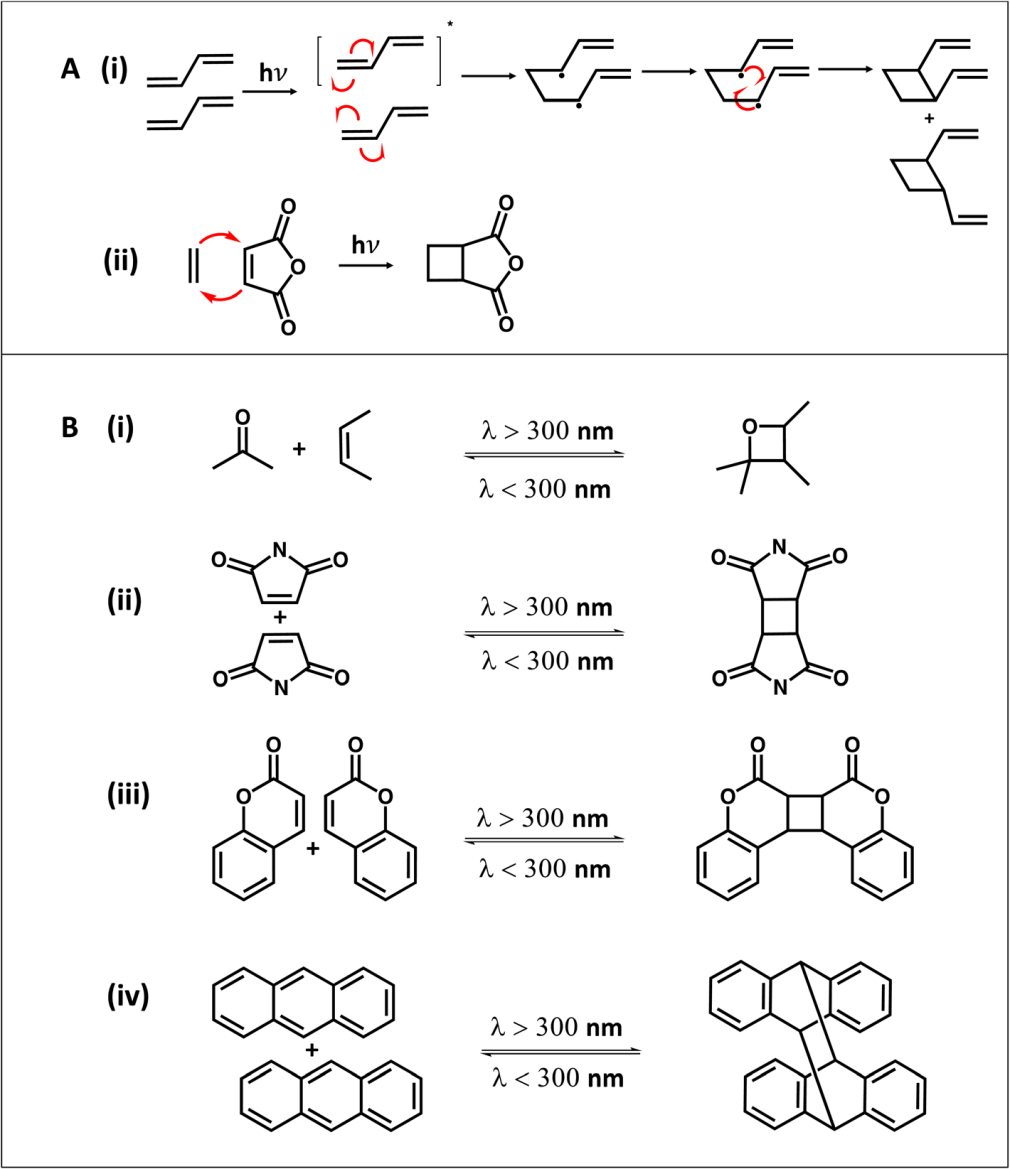
Photoinduced [2 + 2] cycloaddition reaction. (A) (i) Concerted reaction pathway. (ii) Two step radical pathway (adapted from ref. 85 with permission from Elsevier, copyright 2015).^85^ (B) Photoinduced cycloaddition reaction of (i) carbonyl with alkenes,^85^ (ii) maleimide,^94^ (iii) coumarin,^89–91^ and (iv) anthracene.^95,96^

**Fig. 10:**
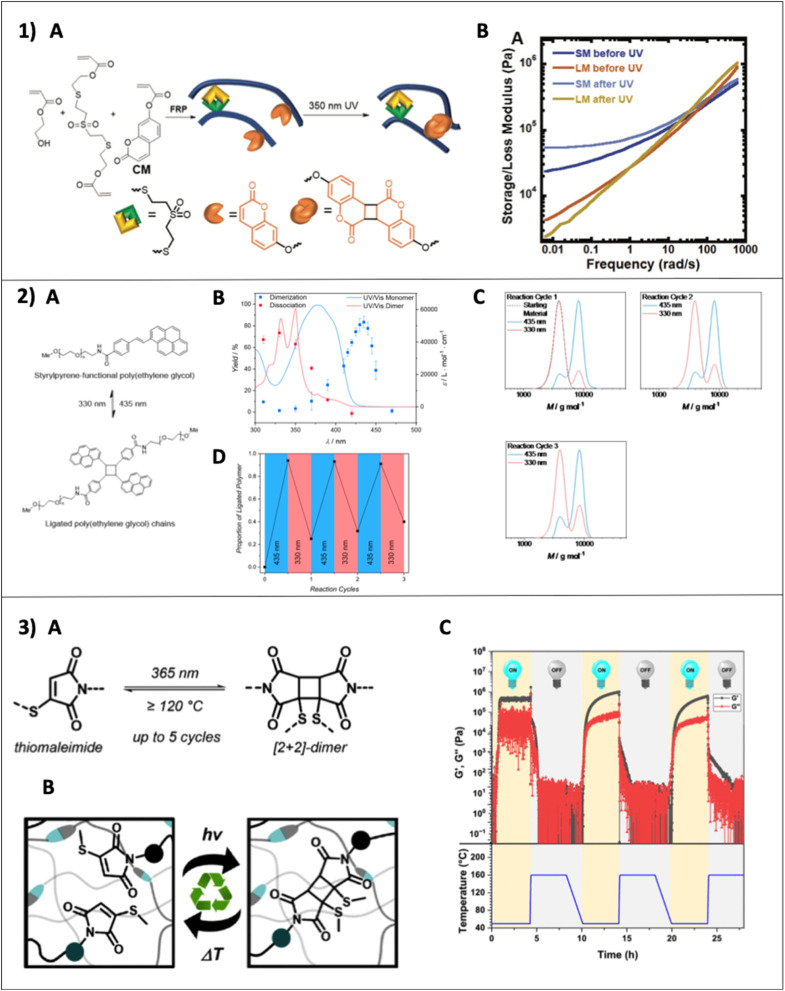
(1) (A) Synthesis of the poly(hydroxy ethyl acrylate) polymer with a TMSDA crosslinker (thiol Michael crosslinker) and coumarin crosslinker (CM). (B) Post-polymerization crosslinking of CM units which undergo [2 + 2] cycloaddition under UVA irradiation (reproduced from ref. 91 with permission from John Wiley and Sons, copyright 2021).^91^ (2) (A) Polymer ligation reaction showing photochemically reversible cycloaddition of *trans*-(*p*-hydroxy)styrylpyrene. (B) Action plot of dimerization and dissociation of *trans*-(*p*-hydroxy)styrylpyrene and its photoproduct mixture. Conditions: irradiation with 242 μmol of photons at each wavelength, wavelength-dependent monochromatic laser irradiation of a 10 mM solution of *trans*-(*p*-hydroxy)styrylpyrene in deuterated acetonitrile. (C) SEC traces of styrylpyrene ligated PEG polymer's dimerization and dissociation products in each reaction cycle. (D) The amount of ligated polymer in reaction cycles with respect to the wavelength (reproduced from ref. 92 with permission from American Chemical Society, copyright 2018).^92^ (3) (A) Photoresponsive forward and thermoresponsive backward reactions of thiomaleimide photodimers. (B) Thiomaleimide photodimers as a responsive dynamic bond in an intrinsically recyclable polymer network. (C) Rheology graph of a bulk network with thiomaleimide crosslinkers under repeating cycles of UV irradiation (*λ* = 365 nm, 7 W cm^−2^, 4 h) and heating (160 °C, 4 h) showing the loss and storage modulus upon crosslinking and decrosslinking (reproduced from ref. 93 with permission from American Chemical Society, copyright 2024).^93^

Cycloaddition reactions occur between two or more π electron systems forming a cyclic adduct.^85^ Two of the most famous cycloaddition reactions are the [4 + 2] cycloaddition and [2 + 2] cycloaddition. Based on frontier orbital theory, the HOMO (highest occupied molecular orbital) of one system interacts with the LUMO (lowest occupied molecular orbital) of the other system and the correct symmetry between the HOMO and LUMO is crucial to accomplish the reaction in both [4 + 2] and [2 + 2] reactions. The classical example for the [4 + 2] cycloaddition reaction is the Diels–Alder reaction under thermal conditions.^86^ However, [2 + 2] cycloaddition reactions can be only accomplished under photochemical conditions, due to it being symmetry forbidden under thermal conditions.^85–87^ A photoinduced [2 + 2] cycloaddition of two unsaturated π systems form a cyclic four membered ring either through a concerted reaction or two step radical pathway as seen in Scheme 4A.^85^ As commonly used reactants in photocycloaddition, coumarins and maleimides undergo [2 + 2] cycloaddition and anthracenes undergo the [4 + 4] cycloaddition reaction under UV light. However, all alkenes do not have the ability to undergo [2 + 2] cycloaddition because of several required parameters needed to construct a cyclobutene including energy of the excited state, or the stability of the excited state. Usually, alkenes with extended conjugation or alkenes with electron withdrawing groups undergo [2 + 2] cycloaddition; therefore there are only a couple of molecules such as coumarin, maleimide, cinnamic acid, thymine, and their derivatives that are useful in cycloaddition reactions.^88^ In addition to the utilization of cycloaddition reactions as a polymerization technique, various cycloaddition reactions have been used in polymer systems as a photodynamic bond to modulate mechanical properties under light or in polymer bioconjugates.^87^ All these motifs are often exploited as photo responsive units in different polymer systems to achieve light dependent higher mechanical properties, self-healing properties, or polymer self-assembly. As seen in Scheme 4B, coumarin undergoes [2 + 2] photoinduced cycloaddition with the ability of undergoing dimerization under light with wavelength *λ* > 300 nm and reversible de-dimerization under wavelength w*λ* < 300 nm,^89^ and is therefore often used as a very attractive component for responsive polymers.^90^ The ability of photofunctionalization of coumarin has been applied in a study by Konkolewicz and coworkers to achieve higher mechanical properties and reconfigurability in a poly(hydroxy ethyl acrylate) polymer after UV irradiation as shown in Fig. 10(1). A two-fold increment of storage modulus has been obtained after UV irradiation in their study indicating the ability of coumarin to be used in light responsive materials.^91^

However, there are several limitations in using cycloaddition reactions in polymer chemistry and the most concerning ones are the necessity of high energy UV light, cycloreversion of the reaction, and the resulting poor yield.^88^ The high energy photons often cause damage to molecules (*e.g.*, denature DNA/proteins or degrade polymers) in the system. Although low yield and cycloreversion can be addressed by adding triplet sanitizers and Lewis acids as a catalyst, they may not be a suitable addition to some systems. Furthermore, alteration of reactivity through structural modifications (*e.g.*, conjugation and electron donating, or withdrawing groups) of the photosensitive group to achieve high yield or to make the systems compatible with relatively low energy visible light, has been explored in recent studies.^92^ Barner-Kowollik and coworkers conducted an investigation of the importance of wavelength dependent behavior of cycloaddition and cycloreversion to minimize spectral overlapping of cycloaddition and cycloreversion to achieve photosensitive reversibility in different applications where re-programmability is needed. Spectral overlap of the forward and backward products of cycloaddition reactions results in a photostationary state or an equilibrium between photoaddition and photocycloreversion. Therefore, spectral decoupling of alkenes and cyclobutene is important. For instance, the styrylpyrene functional group with efficient wavelength dependent photocycloaddition (*λ* = 435 nm) and cycloreversion (*λ* = 330 nm) paths and higher dimerization (95%) yield in cycloaddition and higher dissociation (85%) yield in cycloreversion was coupled with the end of a poly(ethylene glycol) polymer (PEG) to make a ligated polymer system. This system is suitable for biological applications and to develop materials with higher photoreaction control (Fig. 10(2)). Specifically, the system exhibits visible light activation and has shown recyclability for up to three cycles with limited photodamage.^92^ Similar to this, Calvino and coworkers discovered a new *N*-methyl-quinolinone photoresponsive group with a good reaction yield and higher recyclability in [2 + 2] cycloaddition. All properties were also transferred to the PEG polymer coupled *N*-methyl-quinolinone system to achieve efficient photo ligation.^88^

Recently, Houck and coworkers elegantly decoupled forward and backward reactions of [2 + 2] photocycloaddition of thiomaleimide using independent photo activation for bonding and thermal activation above 120 °C for debonding to achieve efficient recyclability in a dynamic crosslinked polymer network as shown in Fig. 10(3A and B). Specifically, not only small molecule study but also the thiomaleimide crosslinker in the network has demonstrated effective forward and backward cycles. Based on rheology data (Fig. 10(3C)), the initial low storage modulus (*G*′) of the bulk polymer network achieved a sudden increment upon UV irradiation because of the bonding of the thiomaleimide crosslinker. Subsequently, upon heating at 160 °C, *G*′ has dropped suddenly because of thermoresponsive debonding of the thiomaleimide dimer, converting the crosslinked network into a viscous solution. Therefore, this strategy has demonstrated an effective way to switch between efficient bonding and debonding states of the thiomaleimide's [2 + 2] cycloaddition reaction.^93^

#### Thiol-X chemistry

**Scheme 5:**
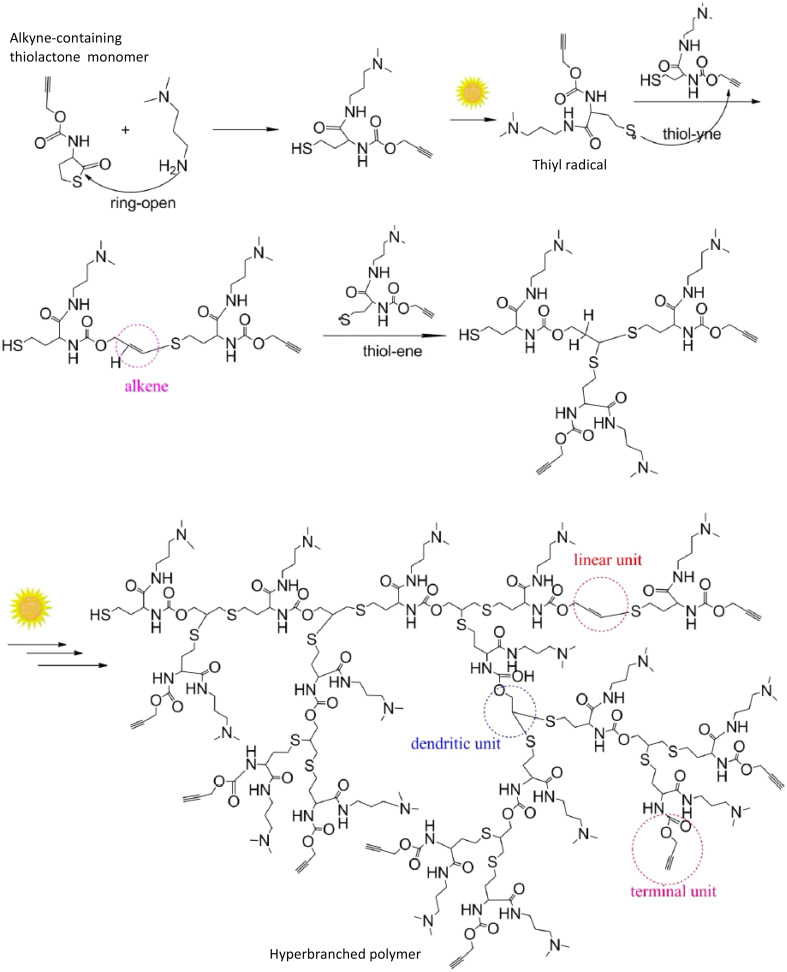
Synthesis of a hyperbranched polymer by polymerizing a monomer with alkyne and thiol groups under sunlight. The monomer with thiol and alkyne groups is generated from the ring opening reaction of a molecule with alkyne and thiolactone groups in the presence of amine (adapted from ref. 104 with permission from Springer Nature, copyright 2013).^104^

Thiol-X chemistry is a robust, versatile coupling reaction of thiols with different functional groups including alkyl halogens, alkenes, or alkynes, to form a C–S bond.^97,98^ Thiol-X coupling reactions are extensively used in a wide range of applications including synthesis and modification of various polymer networks or biomaterials^99,100^ and have been recognized as a “click reaction”. Most commonly thiol–ene, thiol–yne or thiol-Michael reactions are utilized in the synthesis of polymer networks or to introduce different polymer architectures such as hydrogels, dendrimers or hyperbranched polymers.^101–103^ Photoinduced thiol–ene/yne reactions are famous for designing hydrogels (polymerization) or conjugating drug molecules to polymers *via* thiol–ene/yne crosslinkers in various biomedical applications because of excellent controllability and minimal cytotoxicity of light. Besides this, light mediated thiol-X reactions have all the other general advantages of applying light, as discussed throughout the review, including spatial–temporal control, reaction rate modulation, greener energy, *etc.* Yan *et al.* demonstrated the ability of sunlight to activate the thiol-yne reaction to synthesize a hyperbranched polymer using monomers with thiol and alkyne functional groups in the absence of any photocatalyst, as illustrated in Scheme 5.^104^ In this example, sunlight activates the generation of the thiyl radical from the thiol group in the monomer generated *in situ via* a ring opening reaction and this thiyl radical reacts with the alkyne end creating a hyperbranched polymer.

**Scheme 6:**
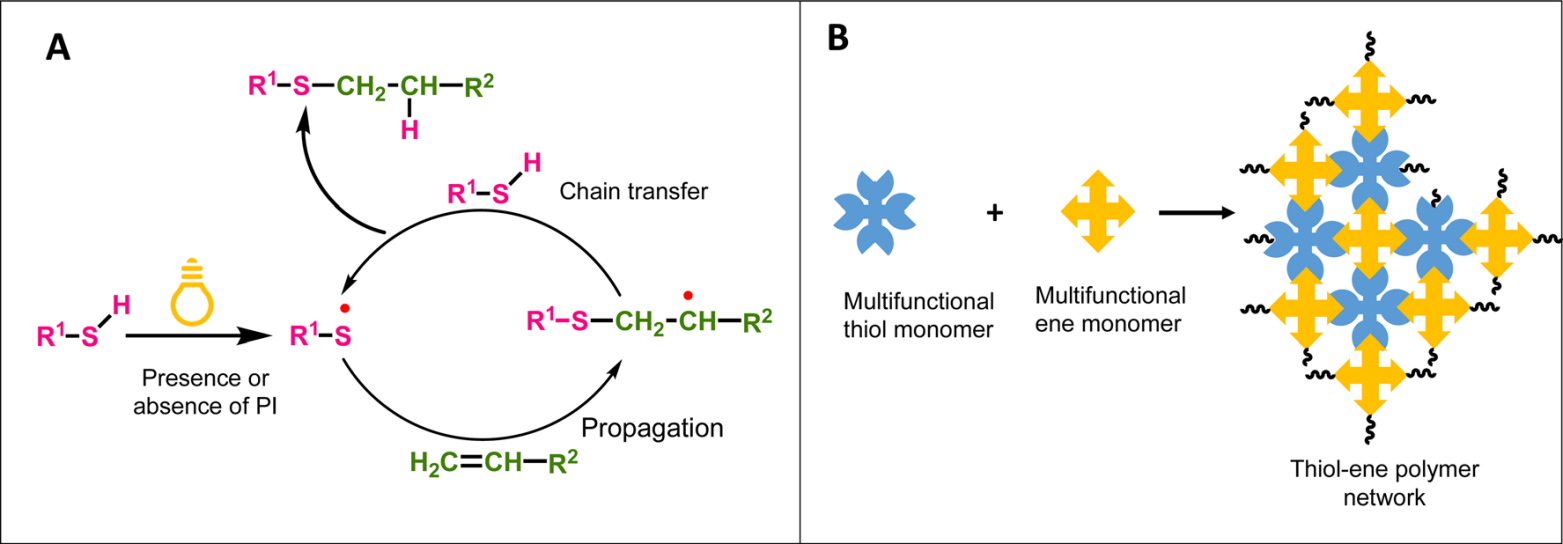
(A) Reaction mechanism of radical mediated thiol–ene photopolymerization under light. (B) Formation of a thiol–ene crosslinked polymer network from multifunctional thiol and ene monomers.

Generally, a radical-based reaction pathway occurs between thiols and electron rich alkenes or alkynes in thiol–ene and thiol–yne reactions, while in thiol-Michael reactions, an anion-based reaction pathway occurs between thiols and an electron deficient alkene.^100^ Radical mediated thiol–ene/yne photopolymerization of multifunctional thiol monomers with multifunctional ene/yne monomers proceeds *via* a step growth pathway to yield a polymer network with a higher crosslink density as illustrated in Scheme 6B.^105^ Furthermore, these highly crosslinked networks show a delayed gel point and homogeneity in the polymer network, in addition to their attractive mechanical properties. Scheme 6A shows the starting of the radical-mediated thiol–ene pathway with the generation of the thiyl radical under light (presence or absence of a radical initiator). This thiyl radical reacts with alkene in the propagation step, creating a new radical center that undergoes chain transfer through H abstraction with thiols reforming a thiyl radical.^106^ Several interesting studies have reported applying light to initiate both radical-mediated thiol–ene/yne polymerization and anion-mediated thiol-Michael polymerization because of having several advantages under light compared to other stimuli such as heat or chemicals (*e.g.*, acids and bases). As shown in Schemes 5 and 6, light generates the thiyl radical in radical mediated thiol–ene/yne polymerization to initiate the polymerization while in thiol-Micheal polymerization, light induces the release of the base or nucleophile to react with the Micheal acceptor and initiate polymerization.

**Fig. 11:**
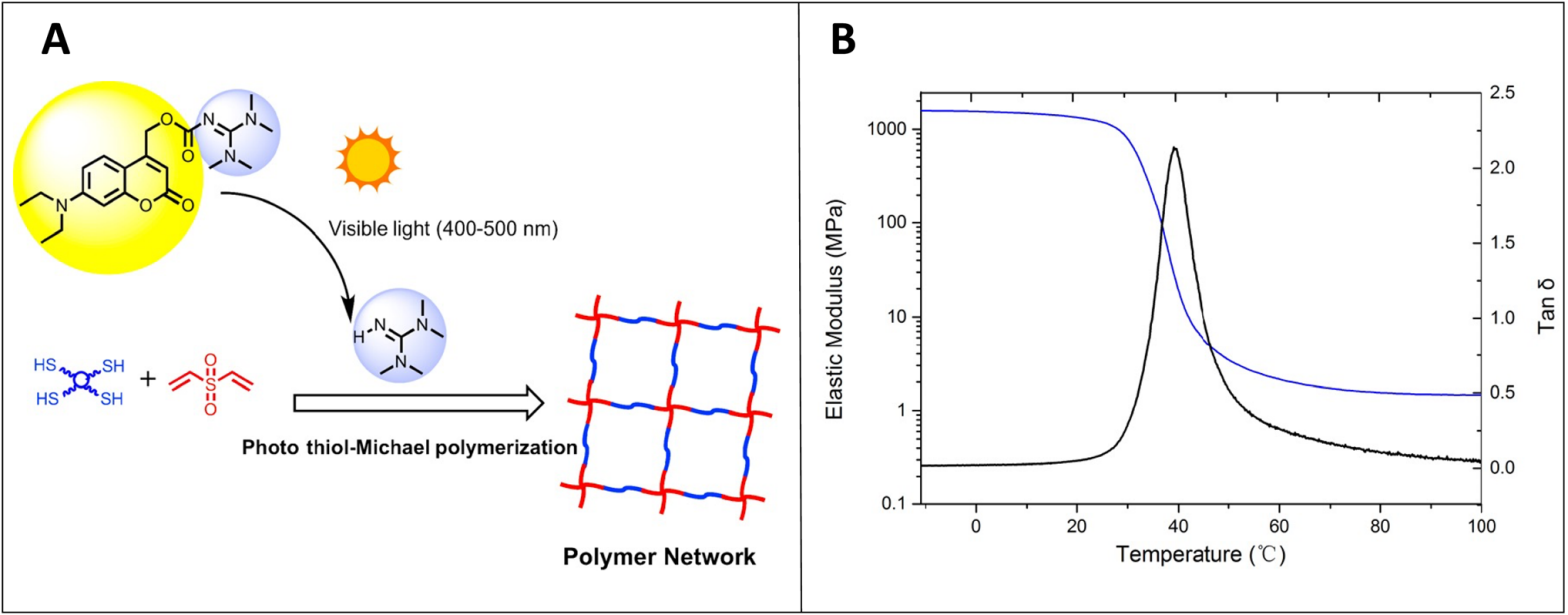
(A) Base generation from the photobase generator under visible light to initiate the thiol-Michael polymerization. (B) Tan *δ* and elastic modulus of the thiol-vinyl crosslinked polymeric film made of PETMP and DVS monomers by using 2 wt% coumarin-TMG with 50 mW cm^−2^, 400–500 nm irradiation (reproduced from ref. 15 with permission from American Chemical Society, copyright 2016).^15^

Bowman and coworkers reported several interesting studies on thiol-X thermal chemistry and thiol-X photochemistry. For instance, they have performed visible light-initiated thiol-Michael addition polymerization in the presence of a highly active photobase generator which is made by coupling a coumarin derivative and a strong tetramethyl guanidine base as depicted in Fig. 11A. The deprotection of the photobase generator under visible light releases the base to initiate the thiol-Michael addition reaction between the tetra thiol monomer (PETMP-pentaerythritol (3-mercaptopropionate) and vinyl monomer (DVS-divinyl sulfone) to generate a homogeneous polymer network. Furthermore, the homogeneity of the synthesized network under light was confirmed by the presence of a relatively narrow tan *δ* peak in the dynamic mechanical analysis (DMA) experiment and the graph is shown in Fig. 11B.^15^

Although the efficiency, ease of implementation, and functional group tolerance of thiol-X chemistry are remarkable, thiol chemistry is always subject to the risk of background oxidation of thiols to disulfides. This runs the risk of perturbing the optimal stoichiometry of reagents or generating undesirable byproducts. However, ongoing work in deoxygenation and oxygen tolerance has the potential to significantly reduce the impact of oxygen-based byproducts on processes that take advantage of thiol X chemistries.

### Photodegradation

#### Photodegradation

**Fig. 12:**
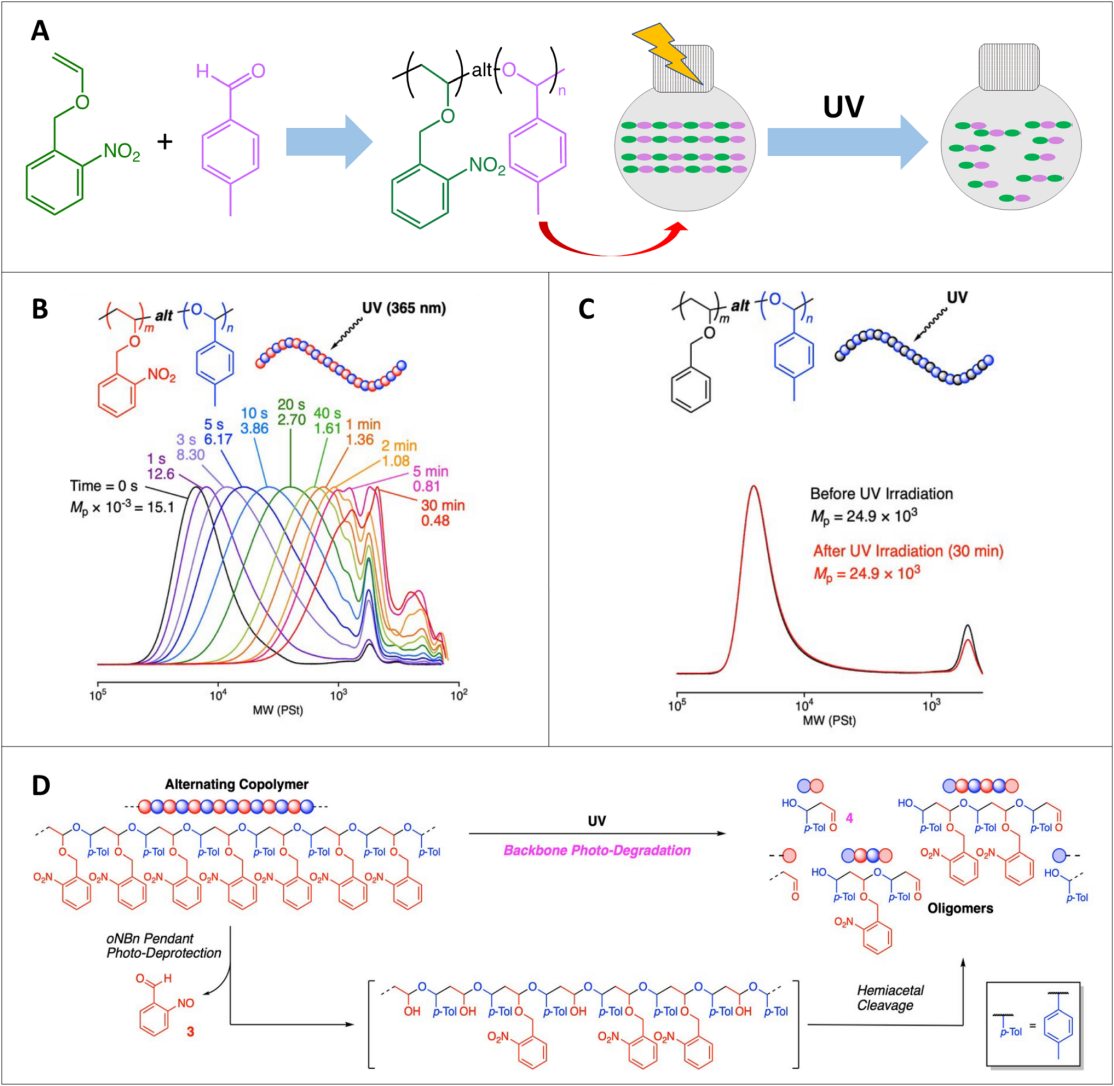
(A) Photodegradation of the poly(*o*NBnVE)-*alt*-poly(*p*MeBzA) alternating copolymer. (B) SEC curves during UV irradiation. (C) SEC curves before and after UV irradiation for the analogous copolymer consisting of BnVE and pMeBzA with no degradation. (D) Photodegradation mechanism of the alternating copolymer of *o*NBnVE and *p*MeBzA (adapted from ref. 126 with permission from John Wiley and Sons, copyright 2023).^126^

Recently, photodegradable polymers have gained interest in two opposite aspects of research and applications including introducing photodegradable properties in a polymer or minimizing the photodegradable properties of a polymer.^107^ When considering photodegradation as an advantage, photodegradable polymers have been studied extensively as a solution for polymer waste management and as a responsive targeted drug delivery system. The accumulation of polymer waste in the environment has been a major concern and using polymer materials that can degrade under light has been recognized as a potential smart solution for that. A wide variety of plastic and polymer materials are being extensively used for short-term or long-term purposes. The estimated worldwide plastic production in 2022 was 400.3 million metric tons and this indicates a huge expansion of plastic usage compared to previous years.^108^ Sadly, this leads to a continuous accumulation of plastic waste in the environment. On the other hand, degradation and aging of short-term plastic and polymer materials under sunlight decreases the mechanical properties and lifespan of the material, and those materials finally end up as waste within a short time period. Therefore, it is crucial to address these problems, and applying degradable materials or non-degradable sustainable materials is interesting. Although using biodegradable,^109,110^ thermally degradable, chemically degradable materials, or traditional waste recyclizing methods,^111,112^ can contribute to addressing these challenges, photodegradable materials^113–116^ are attractive because these materials can degrade gradually under sunlight or other light sources. This is greener compared to chemical or thermal degradation. In the process of photodegradation, molecules/chemical bonds interact with photons and undergo bond dissociation, leading to the formation of small molecules or short oligomers from a polymer chain.^114^ Polymers with photo active groups such as ketones,^113,114^ nitro benzyl groups,^117–120^ coumarins^120,121^ or anthracenes have been used to achieve main chain or side chain cleavage. Recent advances in the field of photodegradable materials have highlighted the potential of vinyl-ketone based polymers. These polymers exhibit rapid degradation under UV irradiation through a process facilitated by the inherent Norrish-type photochemical reactions associated with the ketone moiety side chains of these polymers.^114,122–124^ In particular, homopolymers of phenyl vinyl ketone (PVK) have properties similar to those of polystyrene, which is a commonly used commodity polymer in the production of packaging and films. However, unlike polystyrene, which exhibits prolonged environmental presence even after their usage, because of its slow degradation rates, phenyl vinyl ketone polymers are potentially more environmentally favorable due to their inherent photodegradability. In addition to photodegradable homopolymers, degradable units can be introduced as cleavable side chains or as etchable blocks. A study by Konkolewicz *et al.* found that the incorporation of PVK blocks with BA in the synthesis of thermoplastic elastomers facilitated degradation under simulated ambient sunlight conditions of 1–2 years.^124,125^ In addition to their ability to degrade under light, they have obtained interesting thermomechanical properties in this PVK-BA-PVK thermoplastic material. As demonstrated in Fig. 12A, Ouchi and coworkers designed a copolymer system with photo labile *o*-nitro benzyl pendant groups which rapidly degrade into smaller fragments under UV light, and hence their importance in applications in waste management and targeted delivery.^126^ Interestingly, this alternating copolymer system (poly(*o*NBnVE)-*alt*-poly(*p*MeBzA)) made with an *o*-nitrobenzyl capped vinyl ether monomer (*o*NBnVE) and *p*-tolualdehyde monomer (*p*MeBzA) can withstand ambient light or heat conditions to degrade under light in the absence of any other reactant or conditions. As seen in Fig. 12B below, the molecular weight distribution shifted to a lower molecular weight end during photodegradation, and rapid degradation was achieved within 30 min because of pendent *o*-nitrobenzyl photo-deprotection followed by hemiacetal cleavage.

**Fig. 13:**
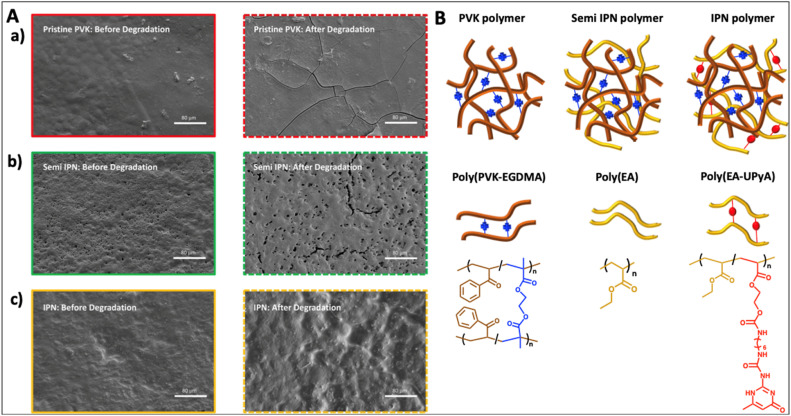
(A) SEM images of (a) pristine PVK materials, (b) semi IPN and, (c) IPN before and after photodegradation. Photodegradation was performed under 350 nm-UV light for 48 h. (B) Structures of a pristine PVK polymer, semi IPN polymer, and IPN polymer (adapted from ref. 125 with permission from Royal Society of Chemistry, copyright 2023).^125^

Recently, controlled photodegradation was reported in PVK polymers which are highly susceptible towards photodegradation through the Norrish mechanism. The degradation under light was controlled by incorporating another polymer network with the PVK polymer and making an interpenetrating network. The formation of a second network increases the opacity and thereby retards the degradation process by preventing light transmittance. Furthermore, SEM images shown in Fig. 13 below reveal a retarded degradation under UV light, showing minimal changes on the IPN surface-(c) and small cracks on the semi IPN surface-(b) compared to propagated network type cracks on the sole PVK surface-(a).^125^ Rapid main chain cleavability of PVK polymers has been proven *via* dramatic *M*_n_ reduction and reduction of glass transition temperature (*T*_g_) upon degradation under light. Furthermore, surface modifications occur as a result of degradation of polymer chains in the whole system.

**Fig. 14:**
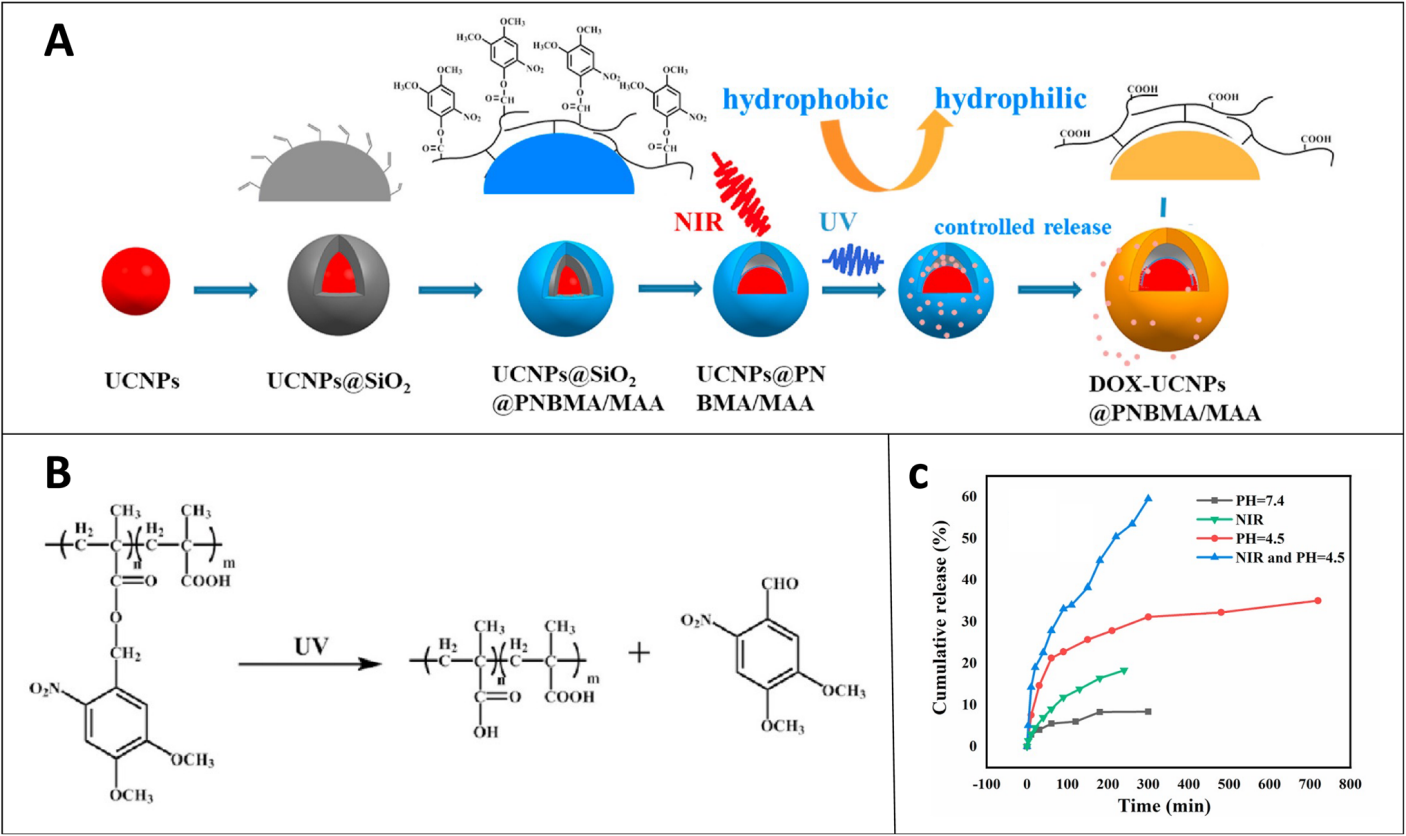
(A) Fabrication of the nanocapsule, loading of the drug and controlled releasing of the drug. (B) Photocleavage of *o*-nitrobenzyl side groups under UV to achieve hydrophobic to hydrophilic transformation. (C) % Cumulative release of DOX from the nanocapsule at pH = 7.4, NIR, pH = 4.5, and NIR-pH = 4.5 (reproduced from ref. 127 with permission from Elsevier, copyright 2021).^127^

In addition to applying photodegradable polymers in waste management, polymers with side or main chain cleavability under light are attractive candidates for a responsive targeted drug carrier because of spatial–temporal control, mouldability, and non-invasiveness. Photoresponsive drug carriers bear photo cleavable groups and release the drug at the targeted site in the presence of an external light stumulus by cleaving photocleavable groups. Therefore, this increases the efficiency of the drug and minimizes undesirable toxic effects on healthy cells. The consideration of light penetration, interaction with cells/biomolecules and phototoxicity needs to be evaluated. Hence, near IR light is often used as an attractive photon source to trigger photoresponsive drug release. A drug vehicle made with polymer nanoparticles with photo cleavable groups such as *o*-nitrobenzyl groups or coumarin groups needs a dramatic disruption of the vehicle to release the drug. Therefore intense/high energy light sources such as UV are required. UV light has a more phototoxic effect on living cells and less ability for tissue penetration. An elegant application was reported by X. Wang and *et al.* using a nanocapsule made with a photo cleavable poly(*o*-nitrobenzyl) shell and lanthanide doped core with upconversion ability to convert UV-shorter wavelengths to NIR-longer wavelengths, thereby making hydrophobic to hydrophilic transformation of the capsule by deprotecting the shell (Fig. 14A and B).^127^ The drug carrier is both photo and pH responsive and, therefore, can release drugs in different environments. Specifically, as seen in Fig. 14C below, ∼60% cumulative release of the doxorubicin model drug was achieved in 300 min in the presence of the synergistic effect of both NIR irradiation and pH 4.5.

Recently, vinyl ketone polymers have been recognized as a potential candidate for target delivery in several studies because of their fascinating photoresponsive behavior; therefore, they can be used in the preparation of different nanocarriers or bioconjugates with photo responsiveness.^123,128^ However, extensive studies including biological systems are essential to understand/broaden their potential and applications further and to evaluate their biocompatibility. This question of the toxicity and impact of byproducts from polymer and organic molecule photodegradation is a larger challenge. The degradation products can also in certain cases be quite strongly colored, such as the products of ONB deprotection, which can inhibit further photochemistry if an appropriate wavelength is not used to avoid this competitive absorption.

#### Photoinduced discoloration and loss of function

Exposure to light can decrease the value, mechanical strength, and lifespan of many polymer materials over time.^116^ Discoloration of a polymer surface, such as yellowing and loss of shine, serves as a key indicator of undesirable reactions occurring within the polymer matrix upon prolonged exposure to light, particularly sunlight and UV radiation. These reactions are often initiated by the absorption of UV light by various additives, chromophores, or impurities present in the polymer, which subsequently trigger photodegradation that results in visible discoloration.^129^ There are two primary causes of color formation in polymers; first, the structural change within the polymer due to side reactions that may occur during manufacturing or in the course of end use. The second is due to the formation of byproducts during the stabilization processes, as stabilizers and antioxidants undergo transformations throughout the lifecycle. The problem of color formation can significantly limit the final application of polymeric products. Color deformation can result from the fundamental oxidation of the polymer itself, or from the ageing of polymers due to the transformation products of hindered phenolics and arylamine antioxidants (thermal antioxidants). These transformation products have been evaluated for their absorption characteristics in the visible spectrum.^130^ Mitigation strategies of discoloration in polymers often involve trapping oxygen-centered radicals, thereby preventing further reactions that could lead to oxidation of the polymers.

In aromatic-based polymers, the presence of benzenoid structures (pro-oxidants) and subsequent oxidation processes can lead to the formation of quinoid compounds, stilbene quinones, and various aromatic carbonyls such as acetophenones, benzophenones, benzoates, vinyl benzoates and aromatic diketones. Additionally, a range of heterocyclic products can be formed, giving rise to a range of distinct colors. The benzenoid structures are often initiators of further oxidation, often by undergoing hydrogen atom abstraction, leading to the formation of hydroperoxides and resulting in significant chain scission and crosslinking.^131–133^ These reactions are also linked to post-operational use, especially under sunlight, aiding color formation.

For instance, polyethylene terephthalate, PET, when exposed to both thermal and UV conditions, undergoes oxidation through hydroperoxidation, where hydroxyl radicals attach to the ring structure, yielding yellow-green colored products.^134–136^ Polycarbonates undergo oxidation primarily under UV light.^137^ Oxidation and crosslinking processes in polycarbonates can generate long-chain benzophenone chromophores, which further induce discoloration through accelerated oxidation. In polystyrene, chromophore formation can occur under both thermal and UV exposure through reactions that generate ketonic and aldehydic products, resulting in color changes.

UV irradiation-induced discoloration is also observed in natural fibers such as wool. Although these fibers are naturally white, prolonged exposure to solar UV radiation results in the degradation of dyes and pigments applied during various applications, leading to discoloration. Consequently, the specific dyes and pigments used play a critical role in determining the extent of discoloration and degradation in these materials.^138^

The degradation process under light is often triggered due to the presence of chromophores in the polymer structure such as ketone or peroxide functional groups, or due to the presence of various chromophore containing impurities, such as solvent, metal or pigments in the matrix.^116^ Common commodity polymers such as poly(styrene) (PSty), poly(vinyl chloride) (PVC), poly(ethylene) (PE) and poly(propylene) (PP) are frequently used outdoors and are prone to photooxidation under light.^116,139^ This process creates radicals in polymer chains on exposure to light which subsequently react with oxygen and create hydroxyperoxyl radicals. Hydroxyperoxyl radicals form hydroperoxides through hydrogen atom abstraction, which then photolyse into hydroxy radicals and alkoxy radicals. The alkoxy radical creates chain scission events, leading into the formation of cracks and making the material lose mechanical integrity and its functionality.^116,139^ Therefore, often various photo stabilizers including quenchers, UV absorbers and radical scavengers are frequently incorporated into polymers to mitigate photodegradation across diverse applications. However, despite their protective role, some of these light stabilizers as well as other additives such as flame retardants are themselves susceptible to photodegradation when exposed to UV radiation. This degradation can further accelerate the deterioration of the polymeric material, ultimately reducing its lifespan.^138,140^ PVC is a widely used plastic in building construction, and it has also found useful outdoor applications such as in sidings or panels. However, upon exposure to UV irradiation, PVC undergoes photodegradation, leading to yellowing and subsequent chalking upon handling.^141^

Similarly, polymeric materials used in devices, such as the backsheet and cable sheath for electricity transport systems and insulation respectively are susceptible to oxidation under solar UV irradiation, causing brittleness.^142,143^ Photovoltaic (PV) devices are sustainable alternative to fossil fuel energy contributing to the reduction in global CO_2_ emission.^144^ These devices consist of multiple layers, including polymeric components like the encapsulant made from ethylene vinyl acetate (EVA) and the backsheet, which is often composed of poly(ethylene terephthalate) (PET) or poly(vinylidene difluoride) (PVDF). Ensuring prolonged service lifetimes with minimal PV failures is essential.^145^ However, solar UV radiation-induced degradation of these polymer components significantly contributes to PV failure as their deterioration can initiate the degradation of other components within the device, so determining the overall lifetime of the PV system.^146^ Furthermore, the yellowing of the backsheet due to UV degradation diminishes the aesthetical value of the PV modules, especially when installed in visible locations like car parks.^141^

Cross-linked poly(ethylene) (XLPE), used as an insulation material in high-voltage cables, also experiences photooxidation when subjected to laboratory-induced radiation in the 350–400 nm wavelength range. This leads to yellowing, chain scission, and the formation of unsaturated groups, which render the surface non-uniform, resulting in fatigue and eventual breakage of the insulating material.^147^ Additionally, the generation of micro- and nanofibers due to UV degradation of textile fibers in certain fabrics further exemplifies the vulnerability of polymers to light exposure.^148,149^

#### Photo enhanced depolymerization

**Fig. 15:**
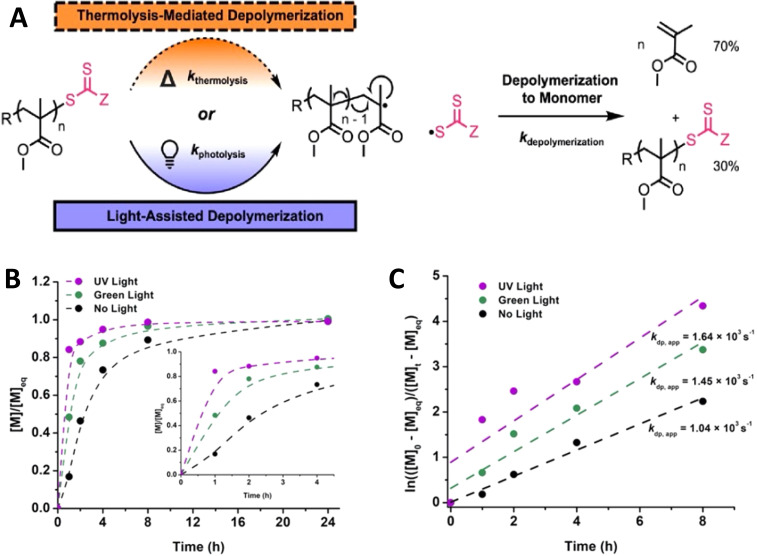
(A) Mechanism of depolymerization of poly(methyl methacrylate) under thermolytic or photolytic initiation. (B) Depolymerization kinetic plots of [*M*]/[*M*]_eq_*vs.* time. (C) Linear pseudo-first order plot showing rates of poly(methyl methacrylate) depolymerization in the presence of dithiobenzoate (reproduced from ref. 150 with permission from American Chemical Society, copyright 2022).^150^

Depolymerization has grown to be a very active area of research in the past few years.^16,150–152^ This has significant sustainability implications since the recovery of monomers is critically important for the post-consumer use of plastics. The key principle in depolymerization is to increase the temperature of the material such that depolymerization begins to dominate over polymerization.^151,153^ By removing volatile monomers such as methyl methacrylate, depolymerization can occur even below the ceiling temperature because of shifting the equilibrium to the forward depolymerization direction favoring more monomer generation. To enhance the depolymerization process, chains can be activated by light to give the same reactive intermediate, such as radicals. This light driven generation of reactive species enhances the rate of depolymerization or equivalently allows a lower temperature at which depolymerization is performed, and especially spatiotemporal control in depolymerization can be achieved.^16^ The vast majority of examples in recent work have focused on radical based methods applied to methacrylic monomers.^150^ Recently, several interesting examples of using light to accelerate depolymerization of RAFT or ATRP-polymers were reported by Sumerlin,^150^ Matyjaszewski,^16^ Anastasaki^16,154^ and coworkers. The Sumerlin group reported photo-assisted radical depolymerization of the methyl methacrylate (poly(MMA)) polymer.^150^ As seen in Fig. 15A below, light initiates the thermal depolymerization process by homolyzing the C–S bond of the RAFT-end group and creating a radical at the end of the polymer chain, which then propagates through the polymer backbone to unzip the polymer chain into methyl methacrylate monomers. Furthermore, kinetic plots in Fig. 15B and C reveal faster depolymerization in the presence of green light and UV light compared to no light conditions. In particular, UV is capable of homolyzing the C–S bond faster and therefore, it increases unzipping, due to the presence of more radicals in the system.

**Fig. 16:**
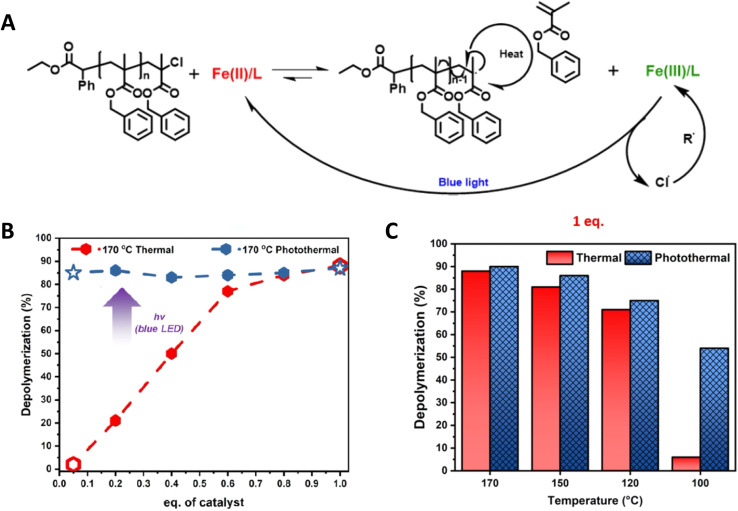
(A) Proposed mechanism of photocatalytic ATRP depolymerization. (B) Comparison of thermal (red) and photothermal (blue) depolymerization of the benzyl methacrylate polymer in the presence of different catalyst loadings. (C) Comparison of thermal and photothermal depolymerization of the benzyl methacrylate polymer at different temperatures in the presence of 1 eq. of the catalyst (reproduced from ref. 16 with permission from American Chemical Society, copyright 2023).^16^

Recently, Matyjaszewski, Anastasaki and coworkers elegantly demonstrated the use of light to lower the depolymerization temperature in poly(benzyl methacrylate) depolymerization synthesized by ATRP. A significant suppression of depolymerization temperature from 170 °C to 100 °C has been achieved under blue light irradiation.^16^ Furthermore, as shown in Fig. 16B and C, remarkable differences in % depolymerization under thermal and photothermal conditions have been obtained at different temperatures using low catalyst concentration. The proposed mechanism is shown in Fig. 16A and based on the fact that the FeCl_2_ catalyst creates an end group radical, which can initiate the depolymerization mechanism and oxidizes into FeCl_3_. Blue light facilitates the reduction of FeCl_3_ into FeCl_2_ to maintain the depolymerization equilibrium.

Although photo enhanced depolymerization has seen significant growth and development over the past 5 years, the most common examples focus on methacrylic polymers. Expansion to other polymer classes, aided by light, is an important area of research and could take advantage of the unique energetic properties of photochemistry to help depolymerize otherwise challenging classes of polymers.

## Conclusions and outlook

Polymers have unique interactions with light. These can sometimes be positive and enable polymers to be generated under unique and mild conditions or enable new transformations. However, at other times, the interactions of polymers with light can be destructive, leading to polymer degradation or enhanced depolymerization. Careful examination of the conditions that drive the light driven generation or destruction of polymers can indeed lead to new properties or new pathways for the end-of-life of plastic-based materials, especially when there is currently a high demand for photo degradable materials. Despite the fact that in the past few years, many studies have introduced photo degradability to undegradable polymers or completely replaced undegradable polymers in a particular application with a suitable photo degradable polymer, a handful of aspects and challenges are yet to be addressed. Mainly, photoresponsive polymers with desirable properties along with photo degradability are important to identify for designing photo degradable polymers in commercial applications. This includes identifying biocompatible photoresponsive polymers as well as light sources in biomedical applications to minimize toxicity. Mainly, the response of many photoresponsive groups in the presence of high energy light sources such as UV light causes negative impacts on cells and tissues. Recent work has shown releasing or degrading polymers using low energy light sources and upconversion techniques in biological systems.^127^ In addition, applying photoremovable protecting groups in biological studies is a very effective technique to achieve precise control as an on–off switch in light induce activation/releasing of biological molecules, and there are several techniques for activation. For instance, those photoremovable protecting groups can be photolyzed with two photon excitations in which two photons are excited simultaneously with NIR light.^155^ This is very effective and highly desirable because of the higher penetration ability of NIR causing minimum photodamage to tissues in contrast to a one photon excitation mechanism which needs higher UV energy. However, further studies are essential to identify more potential candidates and efficient but less harmful photo releasing/degrading mechanisms in biomedical applications.

Faced with the ubiquitous nature of polymers in everyday life,^156^ photochemistry will likely play an important role in addressing their end of life. The unique ability of photochemistry to facilitate challenging reactions such as depolymerization or chain scission could translate from elegant model systems towards the end of life of high molecular weight commodity type polymers. Future studies in applying photodegradable polymers as a solution for polymer waste management should focus on several directions. Common commodity polymers have unique properties such as tensile strength/elongation, compressive strength, hardness, ductility, rigidity, *etc.*^157^ These properties make a particular polymer suitable for a specific application. Therefore, these properties along with degradability under light, make a polymer system a good solution for waste management after their designed purpose. For instance, recently, phenyl vinyl ketone polymers have been recognized as a photodegradable polymer with properties comparable to those of other common commodity polymers like poly(styrene). Degradable blocks or degradable linkers can be introduced into commercial polymers to trigger their degradation under light or sunlight in waste management practices such as recycling or landfilling. This could be done by optimizing the correct amount of photo responsive blocks/linkers in the polymer composition or identifying the proper place to insert them in the polymer architecture. However, these modifications should carefully be engineered to secure desirable properties and durability for the lifetime of the material while having the ability to degrade after its usage. Although lots of studies focus on introducing photo degradable properties to a polymer system, there are only a few studies devoted to understanding the underlying mechanism and identifying photo degraded by products, especially those photo degraded oligomers and small organic molecules that can be reprocessed into highly valuable new materials after proper identification and isolation. Furthermore, most existing photodegradation practices utilize toxic solvents and catalysts. Hence, more environmentally friendly and versatile strategies for direct degradation under light or sunlight are needed.

Coupling depolymerization with light to unzip a polymer under relatively low temperature conditions was studied before.^150^ However, there are lots of further advancements to be made for applying photochemistry in depolymerization. Studies based on photoinduced depolymerization of commodity polymers which are abundantly used on an industrial scale are crucial. These studies need to address scalability issues, optimizing conditions to perform the depolymerization under high concentrations, and under mild light conditions. This is challenging, but with current developments in photochemistry, this is likely to be an ongoing area of research with significant sustainability implications.

The field of polymer photochemistry is continuing to evolve. New applications are being realized, taking advantage of fundamental discoveries to enable applications. Applications that are being actively developed utilize the mild polymerization conditions enabled by polymer photochemistry, for instance in biological and relevant applications. Furthermore, the ability to control where and precisely when polymerization occurs is a significant advantage of polymer photochemistry. This continues to enable new applications in the functional materials space, especially where modification of materials through multiple photochemical processes and complex wavelength dependent chemistry is being developed.^158,159^ Ongoing efforts are expanding beyond the established temporal control, or “on–off” type processes towards complex spatially resolved polymerization needed for 3D printing. A particularly elegant emerging example of love relationships in polymers is the light driven Diels–Alder reaction between naphthalenes and triazolinediones, which require continuous light irradiation to persist as dynamic crosslinks, otherwise the linkages dissociate,^160^ which has implications for temporal and spacial control of polymer crosslinks or 3D printing of vacancies.^161^ Photoinduced functionalization is already being used to generate complex surfaces with resolved properties. Furthermore, with the development of higher intensity and more cost-effective light sources such as applying readily available and cheap direct sunlight, these applications will become substantially simpler to realize and use going into the future.

Even though applying photo response as a polymerization tool is far from new and has been in use for many years in different fundamental research and commercial applications, there is still room for further development and advancement. Mainly, the discovery of a universal photocatalyst with the capability to polymerize any monomer under mild conditions is highly preferred rather than optimizing and synthesizing different photocatalysts in different polymerization systems. In addition to this, advanced computational studies and kinetic modellings would be great additions to understand mechanistic aspects and optimize conditions to achieve better outcomes especially on an industrial scale.

## Author contributions

M. A. S. N. W was involved in writing, editing, conceptualization and visualization. T. N. was involved in writing and editiing. D. K. was involved in conceptualization, writing, editing and supervision.

## Conflicts of interest

The authors declare no competing interests.

## Data Availability

No primary research results, software or code have been included and no new data were generated or analyzed as part of this review.
